# The evolutionary novelty of insect defensins: from bacterial killing to toxin neutralization

**DOI:** 10.1007/s00018-024-05273-5

**Published:** 2024-05-23

**Authors:** Bin Gao, Shunyi Zhu

**Affiliations:** Group of Peptide Biology and Evolution, State Key Laboratory of Integrated Management of Pest Insects and Rodents, Institute of Zoology, Chinese Academy of Sciences, Beijing, China

**Keywords:** Antitoxin peptide, Fold change, Deletion mutation, Gene loss, Gradual evolution, Disulfide bridge reorganization

## Abstract

Insect host defense comprises two complementary dimensions, microbial killing-mediated resistance and microbial toxin neutralization-mediated resilience, both jointly providing protection against pathogen infections. Insect defensins are a class of effectors of innate immunity primarily responsible for resistance to Gram-positive bacteria. Here, we report a newly originated gene from an ancestral defensin via genetic deletion following gene duplication in *Drosophila virilis*, which confers an enhanced resilience to Gram-positive bacterial infection. This gene encodes an 18-mer arginine-rich peptide (termed *Dvir*ARP) with differences from its parent gene in its pattern of expression, structure and function. *Dvir*ARP specifically expresses in *D. virilis* female adults with a constitutive manner. It adopts a novel fold with a 3_10_ helix and a two CXC motif-containing loop stabilized by two disulfide bridges. *Dvir*ARP exhibits no activity on the majority of microorganisms tested and only a weak activity against two Gram-positive bacteria. *Dvir*ARP knockout flies are viable and have no obvious defect in reproductivity but they are more susceptible to the *Dvir*ARP-resistant *Staphylococcus aureus* infection than the wild type files, which can be attributable to its ability in neutralization of the *S. aureus* secreted toxins. Phylogenetic distribution analysis reveals that *Dvir*ARP is restrictedly present in the *Drosophila* subgenus, but independent deletion variations also occur in defensins from the Sophophora subgenus, in support of the evolvability of this class of immune effectors. Our work illustrates for the first time how a duplicate resistance-mediated gene evolves an ability to increase the resilience of a subset of *Drosophila* species against bacterial infection.

**Supplementary Information:**

The online version contains supplementary material available at 10.1007/s00018-024-05273-5.

## Introduction

To combat microbial infection, insects have evolved a set of effective host defense system that operates through humoral and cellular mechanisms to fight against invading microorganisms [1–3]. The former primarily relies on the Toll and immune deficiency (*Imd*) pathways to produce antimicrobial peptides (AMPs) and the latter relies on host haemocyte-mediated phagocytosis, nodulation, encapsulation and melanization [3], all leading to direct microbial killing and infection clearance. The system shows remarkable sophistication in its ability to discriminate among pathogens [4]. For example, in the fruit fly *Drosophila melanogaster*, eight distinct classes of AMPs have been identified, which can be classified in three groups depending on their main microbial targets: (1) Peptides active on Gram-positive bacteria, essentially the defensin; (2) Peptides active on Gram-negative bacteria, including drosocin, cecropins, attacins, diptericins and MPAC (truncated post-translationally modified pro-domain of attacin C); and (3) Peptides active on fungi, including drosomycin and metchnikowin [4]. Using a systematic knockout (KO) approach, Hanson et al. have revealed a synergy and remarkable specificity of these AMPs in vivo [5]. All these microbial killing factors constitute the disease resistance basis of insect host defense.

Additionally, in recent years, the body of evidence supporting the role of peptides in providing disease tolerance (resilience) has been growing. This complementary dimension of host defense allows the host to withstand/tolerate pathogens and repair damages inflicted by the virulence factors of pathogens or the host’s own immune response [6]. Two recent studies revealed the importance of Toll pathway in conferring to resilience of *Drosophila* host defense through the control of the expression of two families of immune-induced peptides (i.e. Bomanins [Boms] and Baramicin A [BaraA]) [6, 7]. The Boms are a family of a dozen secreted peptides that mediate the innate immune response [8]. Deletion of a cluster of 10 *Bom* genes blocks Toll-mediated defenses against a range of fungi and Gram-positive bacteria, which may be due to the *Boms* function in *Drosophila* humoral immunity, mediating direct fungal killing [9]. In addition, specific Boms also can provide protection to the host against the action of fungal toxins to increase its resilience to infection [7]. BaraA is a precursor protein that is cleaved into multiple peptides via furin cleavage sites in response to fungal infection and peptides produced by BaraA exhibit antifungal activity [10]. Moreover, BaraA also protects the fly from the action of distinct toxins secreted by the Gram-positive and fungal pathogens, highlighting a resilience role of these peptides in *Drosophila* host defense [6].

Insect defensins are a group of evolutionarily conserved AMPs of 34–51 residues [11, 12], which belong to the members of the cysteine-stabilized α-helix and β-sheet (CSαβ) superfamily composed of a highly flexible N-terminal loop, an α-helix, followed by an antiparallel β-sheet [13–15]. The α-helix and the second β-strand are linked by two intramolecular disulfide bridges with the third disulfide bridge connecting the N-terminus to the first β-strand [13, 14]. They are classified into the classical insect-type defensins (CITDs) [16]. Since the first discovery in the 1980s in two dipteran insects (*Sarcophaga peregrina* and *Phormia terranovae*), insect defensins have been found in nearly all insect species studied, covering the orders of Diptera, Hymenoptera, Hemiptera, Coleoptera, Lepidoptera, and Phthiraptera, and even exist in mussels and amphioxus [17]. Although several members are identified to have some activity on Gram-negative bacteria and fungi, as key effectors of the innate immune response they primarily mediate resistance on Gram-positive bacterial infections [18]. The antibacterial mechanism of insect defensins is mainly the disorganization of bacterial membranes via the formation of oligomerization surface, causing membrane permeabilization and cell disruption [19]. Using a double stranded RNA (dsRNA) KO approach, Blandin and colleagues have determined the in vivo function of the mosquito *Defensin* and revealed that this peptide is required for the mosquito antimicrobial defense against Gram-positive bacteria [20]. In a prior study, we proposed that an insect defensin-like ancestor may have evolved to a K^+^-channel-targeted neurotoxin (KTx) for scorpion predation and defense [17, 21]. This is achieved by genetic deletion in an evolutionarily variable loop region to remove steric hindrance hampering interactions with K^+^ channels [17], indicative of the evolvability of insect defensins in developing a diverse biological function. The “evolvability’ represents a capacity of a biological system (e.g. organisms or biomolecules) to produce phenotypic variation that is both heritable and adaptive [22, 23].

In the process of studying the evolution of insect defensins among *Drosophila* species, we unexpectedly found a new gene in *D. virilis* that was originated from an insect defensin ancestor via a genetic deletion mechanism. This gene encodes an 18-mer arginine-rich peptide (ARP) with remarkable differences from its parent gene in its pattern of expression, structure and function. Gene KO in combination with in vivo infection experiments unravels its biological role in enhancing the resilience of the fruit fly to Gram-positive bacteria through its toxin neutralization effect, which is complementary to the role of its paralogous defensin that confers the disease resistance through bacterial killing, both jointly providing protection against bacterial infection. Though this gene is restrictedly distributed in the *Drosophila* subgroup with a history of ~ 34 million years ago (MYA), independent deletion variations in insect defensins are also found to occur in the *Sophophora* subgenus, which provides new evidence in support for the evolvability of this class of ancient immune molecules in creating diverse biological functions in different organisms.

## Materials and methods

### PCR primers, peptides, microbial strains and fly colonies

All primers used in this study were synthesized by SBS Genetech (Beijing, China) and listed in Table S1. *Dvir*ARP peptide was chemically synthesized in its reduced form by ChinaPeptides Co., Ltd. (Shanghai, China) with purity > 95%. All microorganisms used in this study and their sources and culture conditions were listed in Table S2. *D. virilis* was gifted from Prof. Qing-Tao Zeng, College of Life Science, Hubei University (Wuhan, China). *D. virilis Dvir*ARP knockout (KO) mutant with frame-shift was created by Fungene Biotech (Beijing, China) and verified by PCR and DNA sequencing.

### Gene discovery

Firstly, using the *Drosophila melanogaster* defensin [GenBank No. NP_523672.1] [11] as query, we searched the wgs contigs database of *Drosophila virilis* via TBLASTN (https://blast.ncbi.nlm.nih.gov/Blast.cgi) with an adjusted expect threshold from the default value (0.05) to 1000, a strategy widely used in our lab to mine distantly related peptides [16]. This search not only led to the identification of the orthologue of the defensin (named *Dvir*DEF), but also to the discovery of a new gene encoding the precursor of an ARP, termed herein *Dvir*ARP. When analyzed the retrieved genomic sequence, we found that this gene is adjacent to *DvirDEF* about 514 bp. Using *Dvir*DEF and *Dvir*ARP as new queries, we systematically searched the *Drosophila* wgs contigs database using TBLASTN to find new defensins and deletion mutants and define their phylogenetic distribution.

### Gene cloning and semi-quantitative RT-PCR

Fifty *D. virilis* adults were put into a sterile flat bottom glass test tube (25 mm x 80 mm) precooled on ice and then poured into a sterile petri dish on ice after 10 min for needle pricking, which was performed by pricking the flies at their dorsal or lateral side of thorax using a thin metal needle. For total RNA extraction, the flies were ground into fine powder in liquid nitrogen and then the RNApure High-purity Total RNA Rapid Extraction Kit (BioTeke Corporation, Beijing) was used to prepare total RNA according to the supplier’s instructions. In brief, the ground sample was first mixed with a highly denaturing guanidine-thiocyanate-containing buffer to inactivate RNases and then ethanol was added. The sample was then applied to an RNApure Mini spin-column, where the total RNA was bound to the membrane and contaminants were efficiently washed away. RNA was eluted in 30 µl DEPC-treated water and stored at -80 °C for use.

For cDNA cloning, total RNAs prepared from the challenged (see above) or not challenged flies were reverse-transcribed into the first-strand cDNAs using RT-PreMix kit (SBS Genetech, Beijing) and a universal oligo(dT)-containing adaptor primer (dT3AP) according to the previously published method [24]. The forward primers used were *Dvir*ARP-F for the *DvirARP* gene and *Dvir*DEF-F for the *DvirDEF* gene and the reverse primer both was 3AP. For genomic cloning, genomic DNA was prepared from *D. virilis* adults and the primers used were the forward primer *Dvir*DEF-F and the reverse primer *Dvir*ARP-R. PCR products were ligated into a T-vector for DNA sequencing (Tsingke Biological Technology, Beijing, China). The primers *Dvir*ARP-F and *Dvir*DEF-F were also used for semi-quantitative RT-PCR via combination with 3AP according to the method described by McPherson and Moller [25]. In this experiment, PCR products obtained from different cycles (25, 30 and 35) were taken for comparison of the amounts of these products. The ribosomal protein  *RP49* gene was chosen as an internal control, which was amplified by the insect RP49 degenerate primer and 3AP from the same cDNA templates.

### Comparative promoter analysis

Proximal and core promoter elements were recognized based on their sequence conservation: insect κB consensus (IUPAC single nucleotide code): GGGRAYYYYY [R = A/G; Y = T/C] [26]; GATA motif consensus: WGATAR [W = A/T; R = A/G] [27]; Initiator (Inr) consensus sequences in *Drosophila*: TCAKTY [A designated as + 1, K = G/T; Y = T/C] for focused promoters and TCA for dispersed promoters [28]. DPE (downstream core promoter element): RGWYV [W = A/T; R = A/G; Y = T/C; V = A/C/G] [29]; TATA box: TATAWR [W = A/T; R = A/G] [30].

### Structural modeling

To build a three-dimensional (3D) structure of *Dvir*ARP, the Fugue tool (a sequence-structure homology method using environment-specific substitution tables and structure-dependent gap penalties) was firstly employed to find suitable templates for comparative modeling [31]. This method identified the experimental structure of MSL2 CXC (residues 24–41) (PDB entry: 4RKH) as the best template, which was used to create the 3D model of *Dvir*ARP with Modeller V9 [32, 33]. The MSL2 is a male-specific lethal (MSL) complex protein involved in dosage compensation process of male *Drosophila* [34]. The model was evaluated by the QMEAN [35] with a score of 0.258. The model of *Dtri*DLP-1 was built via trRosetta with restraints from both deep learning and homologous templates. The confidence of the model is very high (Estimated TM-score: 0.823). Models of *Dtri*TM(CX5C) and *Dtri*TM(CX8C) were also built via trRosetta but only based on *de novo* folding, guided by deep learning restraints due to the lack of templates and thus showing a low confidence.

To build the complex model of *Dvir*ARP and a DNA structure, we used a template-based structure modeling method to replace the CXC domain in its complex with a DNA sequence and the resultant *Dvir*ARP-DNA model was energetically minimized by MOE (Molecular Operating Environment) (https://www.chemcomp.com/Products.htm) using AMBER14, an all atom force field for simulations of proteins and nucleic acids. Secondary structure assignment from the atomic coordinates of *Dvir*ARP was performed with STRIDE (http://webclu.bio.wzw.tum.de/stride).

### Prediction of cation–π interactions

Cation–π interactions within the *Dvir*ARP structure were predicted with the CaPTURE program written by Justin Gallivan [36] (http://capture.caltech.edu/), in which E_es_ and E_vdW_ mean electrostatic and van der Waals interactions, respectively.

### Evolutionary tree construction

The evolutionary tree of the *Drosophila RP49* gene was inferred using the Neighbor-Joining method. The evolutionary distances were computed using the p-distance method and are in the units of the number of base differences per site. This analysis involved 31 nucleotide sequences. All ambiguous positions were removed for each sequence pair (pairwise deletion option). There were a total of 410 positions in the final dataset. Evolutionary analyses were conducted in MEGA11 [37]. *Drosophila* RP49 gene sequences and accession numbers are provided in Appendix 1.

### Oxidative refolding of chemically synthesized *Dvir*ARP

The oxidative refolding of synthetic *Dvir*ARP was performed according to the method previously described [17, 38]. Briefly, the synthetic peptide was firstly dissolved in water in a concentration of 2 mg/mL and then 100 mM Tris-HCl (pH 8.0–8.5) was used to dilute the peptide solution to a final concentration of 0.1 mg/mL. The solution was incubated for 24 h at 25 °C and the oxidized product was then purified by reversed-phase high-performance liquid chromatography (RP-HPLC). The collected peak was lyophilized by Thermo Scientific SAVANT SPD1010 SpeedVac Concentrator (USA). The purity and molecular mass was identified by matrix-assisted laser desorption/ionization time of flight mass spectrometry (MALDI-TOF MS) using an Ultraflextreme^™^ instrument (Bruker Daltonics, Bremen, Germany) in a positive-ion mode and α-cyano-4-hydroxycinnamic acid (CHCA) as a liquid matrix. Molecular weights (MWs) of peptides were calculated with Protein Calculator v3.4 (https://protcalc.sourceforge.net/).

### Assignment of disulfide bridges in *Dvir*ARP

Oxidized *Dvir*ARP (50 µg) was incubated with trypsin at a peptide/enzyme ratio of 50:1. Digestion was performed in a buffer containing 50 mM Tris-HCl (pH 8.0) and 1 mM CaCl_2_ for 16 h at 37 °C. The digestion was stopped by acidification with 0.05% TFA. Separation of peptide fragments was performed onto a Zorbax 300SB-C18 (4.6 × 150 mm, 5 μm) column (Agilent, USA) with a linear gradient from 0 to 60% acetonitrile in 0.05% TFA within 40 min with a flow rate of 1 mL/min. Two fragments (named P1 and P2) were analyzed by MALDI-TOF MS.

### Circular dichroism (CD) spectroscopy

For CD analysis, a peptide sample was dissolved in 5 mM phosphate buffer (PB, pH7.0) with a concentration of 0.1–0.2 mg/mL. CD spectra were measured on the Chirascan Plus spectropolarimeter v.4.4.0 (Applied Photophysics Ltd, UK) by using a quartz cell of 1.0 mm thickness. The wavelengths used ranged from 190 to 260 nm. Data were collected at 1 nm intervals with a scan rate of 0.5 s per point and expressed as delta epsilon (cm^-1^M^-1^). Delta epsilon was calculated as [θ×(MRW×0.1)/(C×L)/3298], where θ is the ellipticity (in millidegrees), C is the concentration (in mg/mL), L is the path length (in cm), and MRW is the mean residue weight (in Da).

### Antimicrobial assay

An inhibition-zone assay [38] was used to quantify the antibacterial activity of *Dvir*ARP. In brief, an overnight bacterial culture from a single colony was inoculated into fresh medium and grew to late log-phase. A 10 µL aliquot of each culture was diluted in 6 mL pre-heated medium containing 0.8% agar. The mixture was spread on a 9-cm Petri dish, giving a depth of 1 mm. After settling, 2-mm wells were punched in the plate and then 2 µL peptide samples of different concentrations were added to each well. The peptide was dissolved in 5 mM PB buffer (pH 7.0) unless otherwise indicated. The agar plates were incubated overnight at indicated temperatures. A lethal concentration (C_*L*_) was calculated from a plot of *d*^2^ against log *n*, where *d* is the diameter (in cm) and *n* is the amount of sample applied in the well (in nmol). The plot is linear and thus C_*L*_ can be calculated from the slope (k) and the intercept (m) of this plot. The formula used here is C_*L*_=2.93/ak10^m/k^, where a is the thickness of the bacterial plate in cm (0.1) and C_*L*_ is in µM. A similar procedure was used to evaluate the activity of the peptide against the fungi, in which spore suspension were harvested and used to prepare plates for antifungal assay [39].

### Electrophoretic mobility shift assay (EMSA)

Five µM of annealed double strand S15 (5’-ATGAGCGAGATGGAT-3’) and different concentrations of *Dvir*ARP (2, 4 or 8 µM) were incubated at room temperature for 20 min in a 20 µL of binding buffer containing 4% glycerol, 1 mM MgCl_2_, 10 mM NaCl, 0.5 mM EDTA, 0.5 mM DTT, and 10 mM Tris-HCl pH 7.5. The mixtures then were resolved in 6% native polyacrylamide with TBE buffer (45 mM Tris-HCl pH 8.0, 45 mM boric acid, 1 mM EDTA, 200 V 20 min). DNA-protein complexes were detected by ethidium bromide staining. Recombinant mouse FOXN1 DNA binding domain (mFOXN1 DBD) and the specific binding motif *Mm*MCM2 (*Mus musculus mini-chromosome maintenance protein* gene) (5’-CCTTAGCGTGGTAA-3’) was used as a control of this method.

### *Dvir*ARP knockout

For *DvirARP* knockout, sgRNA targets were firstly designed with CRISPR Optimal Target Finder (http://tools.flycrispr.molbio.wisc.edu/targetFinder/) [40]. The template for in vitro transcription by T7 polymerase was generated by annealing of two DNA oligonucleotides and PCR amplification. In vitro transcription was performed with the T7 RiboMAX^™^ Kit (Promega, P1320). In this experiment, two sgRNAs were designed to target the coding DNA sequence (CDS) about 25 ~ 70 bp downstream of the start codon which can bring frame-shift to the gene. Plasmid MLM3613 (Addgene plasmid 42,251) was linearized with Pme I (New England Biolabs) and purified by ethanol precipitation. Cas9 mRNA was transcribed with mMESSAGE mMACHINE® T7 Transcription Kit (Ambion), polyadenylated with the *Escherichia coli* Poly(A) polymerase Kit (NEB), and purified with the RNeasy Mini Kit (QIAGEN). 15 µg of Cas9 mRNA, 7.5 µg sgRNA were mixed with DEPC water in a 30 µL volume. Embryos were injected using standard protocols. Injections were carried out at 18 °C and embryos were shifted to 25 °C immediately following injection. When the P0 and F1 flies grew into adults, they were crossed with *D. virilis*. The genomic DNA of the P0 and F1 flies was extracted. PCR was performed using primers flanking the target region. Amplified products were purified for Sanger sequencing to valid the frame-shifted small insertion or deletion. The flies with homozygous mutation was obtained.

### Pupation assay of flies

Laboratory culture of *Drosophila* was performed according to the method of Michael Ashburner and John Roote [41]. Each pair of flies (a naive male and a virgin female) were loaded into a 100-mL Erlenmeyer flask with 2-cm-thickness medium and cultured at 26 °C. In this condition, time to pupation was about 13 days for both the wild-type and mutant flies. The experiment lasted for 20 days. Each group was tested with five replicates for the wild-type and mutant flies. Each pupal number was recorded and compared between the wild-type and mutant with Student’s t-test.

### Systemic infections and fly survival

Systemic infection experiments were performed accoroding to the methods decribed previously [5–7]. In brief, the bacteria *Staphylococcus aureus* CGMCC 1.89 and *E. coli* DH5α were seperately cultured to OD_600_ = 0.4 with broth and LB media at 37 °C, respectively. After pelleted by centrifugation, the bacteria were washed twice by phosphate-buffered saline (PBS, pH 7.5) and then resuspended in PBS to the same concentration. *D. virilis* adult females, both wild-type (*Dv*WT) and *DvirARP* mutant (*Dv*delARP) were anesthetized on ice and injected into thorax with 69 nL of the bacterial suspension or PBS for each fly with the Nanoliter 2010 microinjection system (WPI, USA). Infected flies were subsequently maintained at 26 °C for experiments. Dead flies were daily counted over a period of seven days, in which flies that died within 24 h of injection were excluded in the analysis. Seventy flies per condition were used in this study.

### Preparation and injection of *S. Aureus* supernatant and fly survival

Twelve mL of overnight culture of *S. aureus* CGMCC 1.89 grown in broth medium at 37 °C were collected and centrifuged; the supernatant was then filter-sterilized by passing through a 0.22-µm-pore-size sterile syringe filter. The sterilized supernatant was concentrated 15-folds by ultrafiltration [CBU(15x)] in a 3-kDa cut-off Amicon Centricon filter (MWCO 3 kD, Millipore) to collect the molecules larger than 3 kDa. The CBU(15x) was then injected into flies (*Dv*WT and *Dv*delARP) with a dose of 69 nL each fly with the Nanoliter 2010 microinjection system (WPI, USA). Injected flies were subsequently maintained at 26 °C for experiments. Dead flies were daily counted over a period of seven days. Seventy flies per condition were used in this study. We also prepared a 15x concentrated sample through lyophilization by vacuum freeze-drying and resuspended with water but found that it lost some components relative to the original supernatant, as identified by sodium dodecyl sulfate polyacrylamide gel electrophoresis (SDS-PAGE) analysis, and thus discarded it in our study.

### Statistics

Statistical analysis of pupation numbers between *Dv*WT and *Dv*delARP flies was carried out using SPSS Statistics 17.0 (SPSS Inc.). Data are expressed as mean ± standard deviation (SD) (*n* = 5). Statistical significance of means between two groups was determined by unpaired two-tailed Student’s t-test for the data that accorded with normal distribution and homogeneity of variance. Direct comparisons of the survival ratios between *Dv*WT and *Dv*delARP were performed using Log-rank (Mantel-Cox) test and Gehan-Breslow-Wilcoxon test in GraphPad Prism 8.02. Generally, *P* > 0.05 was considered statistically insignificant (ns), *P <* 0.01 was considered significant (**), *P <* 0.001 was considered highly significant (***), and *P <* 0.0001 was considered very highly significant (****).

## Results

### *Dvir*ARP, an ARP, is evolutionarily related to insect defensin in *D. Virilis*

**Fig. 1:**
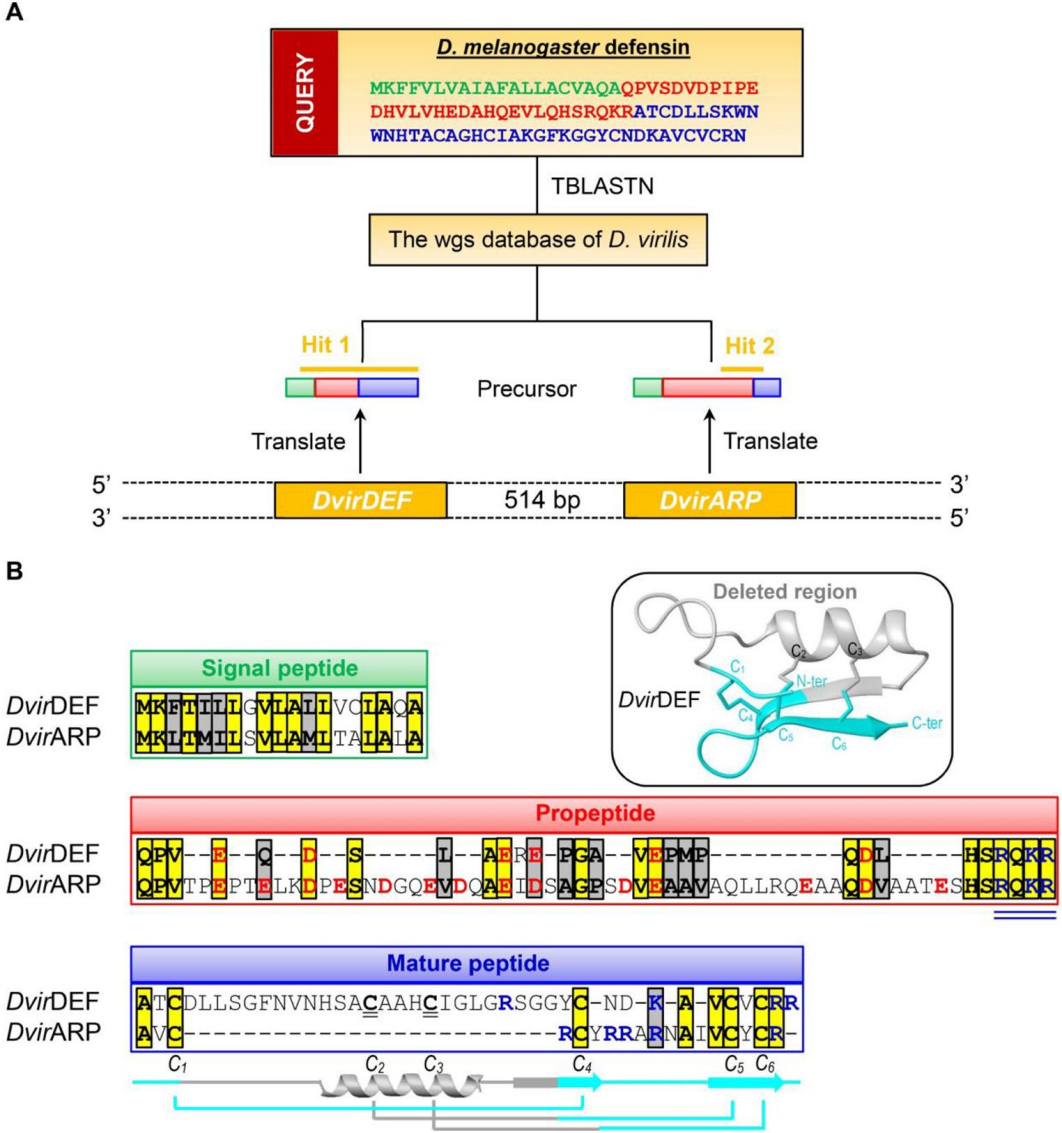
Gene discovery of *Dvir*ARP and its similarity to *Dvir*DEF. (**A**) TBLASTN-based gene discovery strategy. *D. melanogaster* defensin was used as query to search the wgs database of *D. virilis*, which led to the discovery of the *DvirDEF*–*DvirARP* cluster on the chromosome. The regions in two hits detected by TBLASTN sharing similarity with the query are indicated by orange lines. (**B**) Precursor sequence comparison. Signal peptides, propeptides and mature peptides are boxed in *green*, *red* and *blue*, respectively, in which identical and conserved residues are boxed and shadowed in *yellow* and *grey*, respectively; acidic (D or E) and basic (K or R) residues shown in *red* and *blue*, respectively in the propeptide and the mature regions. In the end of the propeptide region, a Kex2-like processing endoprotease cleavage motif (RQKR) is underlined twice in *blue*. Two evolutionarily deleted cysteine residues in *Dvir*DEF are underlined twice. Secondary structure elements and the disulfide bridges of *Dvir*DEF are extracted from its computational model. Inset, the ribbon structure of *Dvir*DEF highlighting the evolutionarily deleted region in an insect defensin (in *grey*) and the remaining part evolving into ARP (in *cyan*)

By applying a TBLASTN-based gene discovery strategy, we searched the whole-genome shotgun contigs (wgs) database of *D. virilis* using the *D. melanogaster* defensin (*Dmel*DEF) precursor [4, 11] as query (Fig. 1A), which led to the discovery of the defensin ortholog (named *Dvir*DEF) and an adjacent gene encoding the precursor of a new peptide (herein named *D. virilis* arginine-rich peptide (abbreviated as *Dvir*ARP) given its high content of arginine residues) (Fig. 1B). Figure 1A shows the similarity region detected by TBLASTN between precursors of *Dvir*ARP/*Dvir*DEF and *Dmel*DEF (Fig. 1A). These two *D. virilis* genes comprise a gene cluster on the.

chromosome with a distance of 514 bp (Fig. 1A). The precursor of *Dvir*ARP consists of 98 amino acids and is composed of three distinct domains: an amino-terminal signal peptide of 19 residues followed by a propeptide of 61 residues rich in negatively charged residues (6 Asp and 8 Glu) terminated by a Kex2-like processing endoprotease cleavage motif (RQKR) [42], and a carboxyl-terminal part comprising the mature peptide of 18 residues rich in arginine residues (Fig. 1B).

Though TBLASTN only detected a small portion of sequence similarity between the insect defensin and *Dvir*ARP, a closer examination of the precursor sequences of these two *D. virilis* peptides revealed three commonalities: (1) They share a completely identical precursor organization comprising a hydrophobic residue-rich signal peptide, an acidic residue-rich propeptide and a mature peptide; (2) They share detectable sequence similarity in the signal peptide and propeptide region, especially with completely identical processing signals, namely the cleavage of signal peptides between an Ala and a Qln and the cleavage of propeptides between the recognition motif (RQKR) and an Ala; (3) They share 50% sequence similarity in the mature peptide region, including four strictly conserved cysteine residues, two Ala residues, one Val and two basic residues (Fig. 1B). Therefore, the new peptide can be considered as a naturally-occurring deletion mutant of insect defensins, in which the region spanning the N-terminal loop and the α-helix together with partial β1 strand was removed in evolution (Fig. 1B, inset). Next, we conducted a series of experiments to assess the transcriptional feature, the structure and biological functions of *Dvir*ARP to illustrate how a newly originated gene confers an enhanced immune defense function to its host.

### Female-specific and constitutive expression of *DvirARP*

**Fig. 2:**
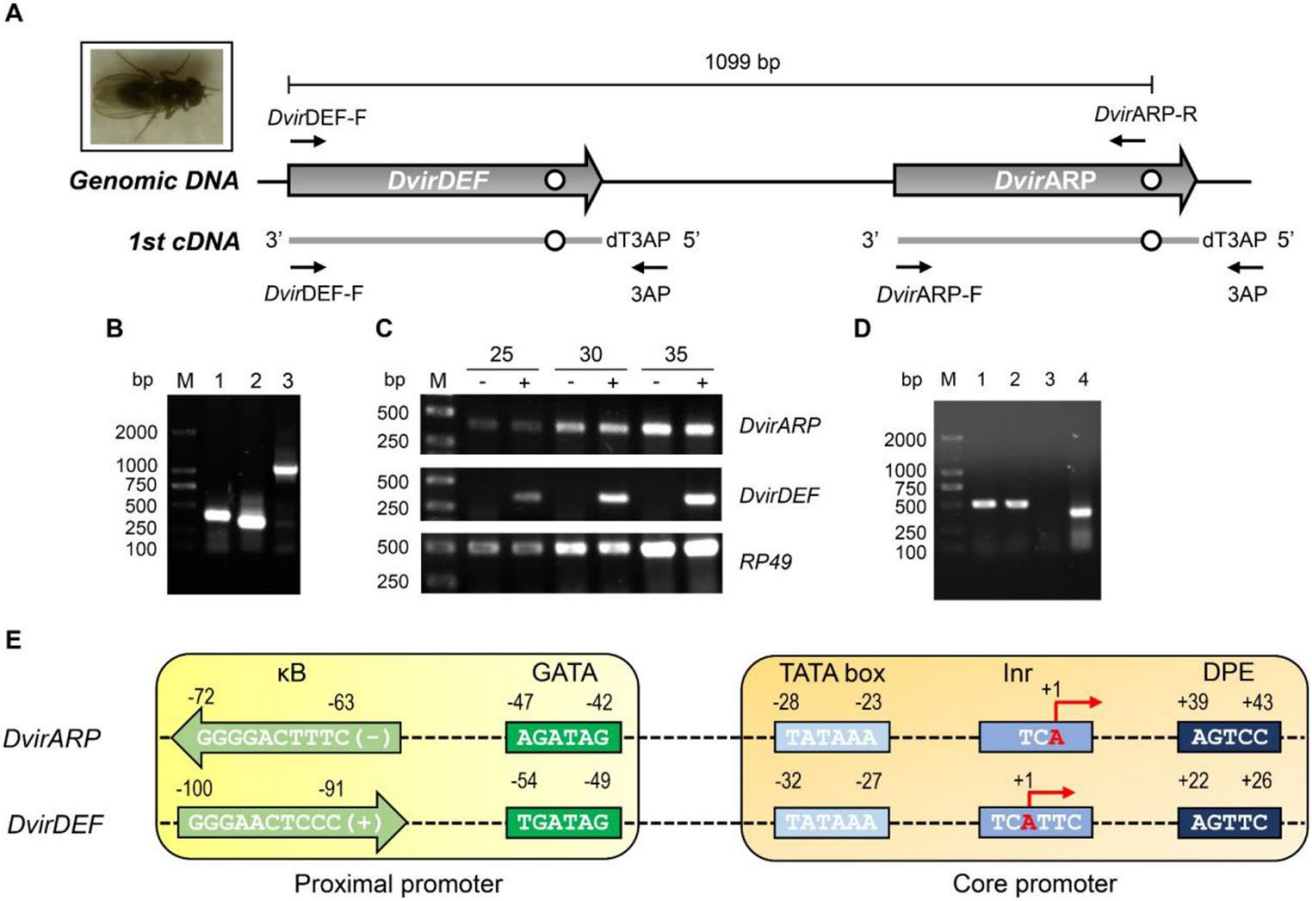
Identification of *DvirARP* and *DvirDEF* at the transcriptional level. (**A**) Schematic drawing depicting the *DvirDEF - DvirARP* cluster and PCR primers and their positions on the genomic DNA and the first-strand cDNA. (**B**) Amplification of *DvirARP* (lane 1) and *DvirDEF* (lane 2) from the cDNA template prepared from *D. virilis* adults challenged by pricking and of the genomic region of *DvirDEF* and *DvirARP* with primers *Dvir*DEF-F and *Dvir*ARP-R. (**C**) Semi-quantitative RT-PCR detecting the expression patterns of *DvirARP* and *DvirDEF* genes in *D. virilis* adults without (“-”) and with (“+”) needle pricking. *RP49* was used as internal control. (**D**) Sex-specific expression of *DvirARP*. Lanes 1 and 2: amplification of the *RP49* cDNA from male and female adults, respectively; Lanes 3 and 4: amplification of *DvirARP* cDNA from male and female adults, respectively. Lane M: DNA marker. (**E**) Proximal and core promoter elements of *DvirARP* and *DvirDEF* genes. The locations of the sequence motifs are roughly to scale

Using PCR and DNA sequencing techniques, we verified the sequences of the *DvirDEF* - *DvirARP* gene cluster in the *D. virilis* genome and their cDNA sequences amplified from the cDNA template prepared from the *D. virilis* adults challenged by pricking to mimic fly injury [43] (Fig. 2A and B). Semi-quantitative RT-PCR showed that the two genes displayed a different expression pattern, inducible expression in *DvirDEF* and constitutive expression in *DvirARP* (Fig. 2C). Moreover, the *DvirARP* cDNA was only amplified from the template prepared from the female other than male adults (Fig. 2D), indicating that *DvirARP* is a female-specifically expressed gene in *D. virilis* adults.

To study the molecular basis responsible for the expression pattern differential between the two genes, we carried out comparative promoter analysis of their promoter regions. It is found that they both had an overall conserved regulatory motifs in their proximal and core promoter elements (see Fig. S1), which included a κB- like motif [GGGGACTTTC(-) in *DvirARP* and GGGAACTCCC(+) in *DvirDEF*] and a GATA motif [AGATAG in *DvirARP* and TGATAG in *DvirDEF*] in their proximal promoters; and three regulatory motifs in their core promoters [TATA box, Inr and DPE] (Fig. 2E). A large number of insect immunity genes contain a GATA motif situated close to the κB motif in their regulatory regions [44, 45] and they both have been shown to be necessary for full *Drosophila Cecropin A1* (*CecA1*) promoter.

activity in transfection assays [26, 27]. Hence, the presence of these two motifs in their proximal promoter can well account for the inducible expression pattern of *DvirDEF* but could not intuitively explain the pattern of *DvirARP*. We found that in spite of the overall conservation, there still exist subtle differences between the *DvirDEF* and *DvirARP* promoters: (1) κB-like motif orientation. It is known that regulatory motifs can be found in two possible orientations relative to gene direction: namely the transcribed template (3’ to 5’) and the non-template (coding strand) (5’ to 3’). Similar to other inducible insect immune genes, the κB in *DvirDEF* is oriented in a coding strand direction (+) whereas the κB in *DvirARP* is in a template orientation (-) (Fig. 2E). Since transcription factor binding site (TFBS) orientation and order are major drivers of gene regulatory activity [46], it is reasonable to infer that the orientation difference in κB could determine their expression pattern differential. (2) This is further reinforced by their Inr element, a core promoter element encompassing the transcription start site. The Inr of *DvirDEF* is TCA(+ 1)TTC [A designated as + 1] whereas that of *DvirARP* is TCA(+ 1) (Fig. 2E). It has been found that in eukaryotic transcription start sites, there is a general correlation of focused transcription with regulated genes that have an Inr of TCA(+ 1)KTY [K = G/T; Y = T/C] and dispersed transcription with housekeeping genes that have an Inr of TCA(+ 1) [28]. This analysis provides additional support for *DvirDEF* as a regulated gene and *Dvir*ARP as a housekeeping gene that typically has a steady level of transcription.

### *Dvir*ARP is a disulfide-linked peptide

**Fig. 3:**
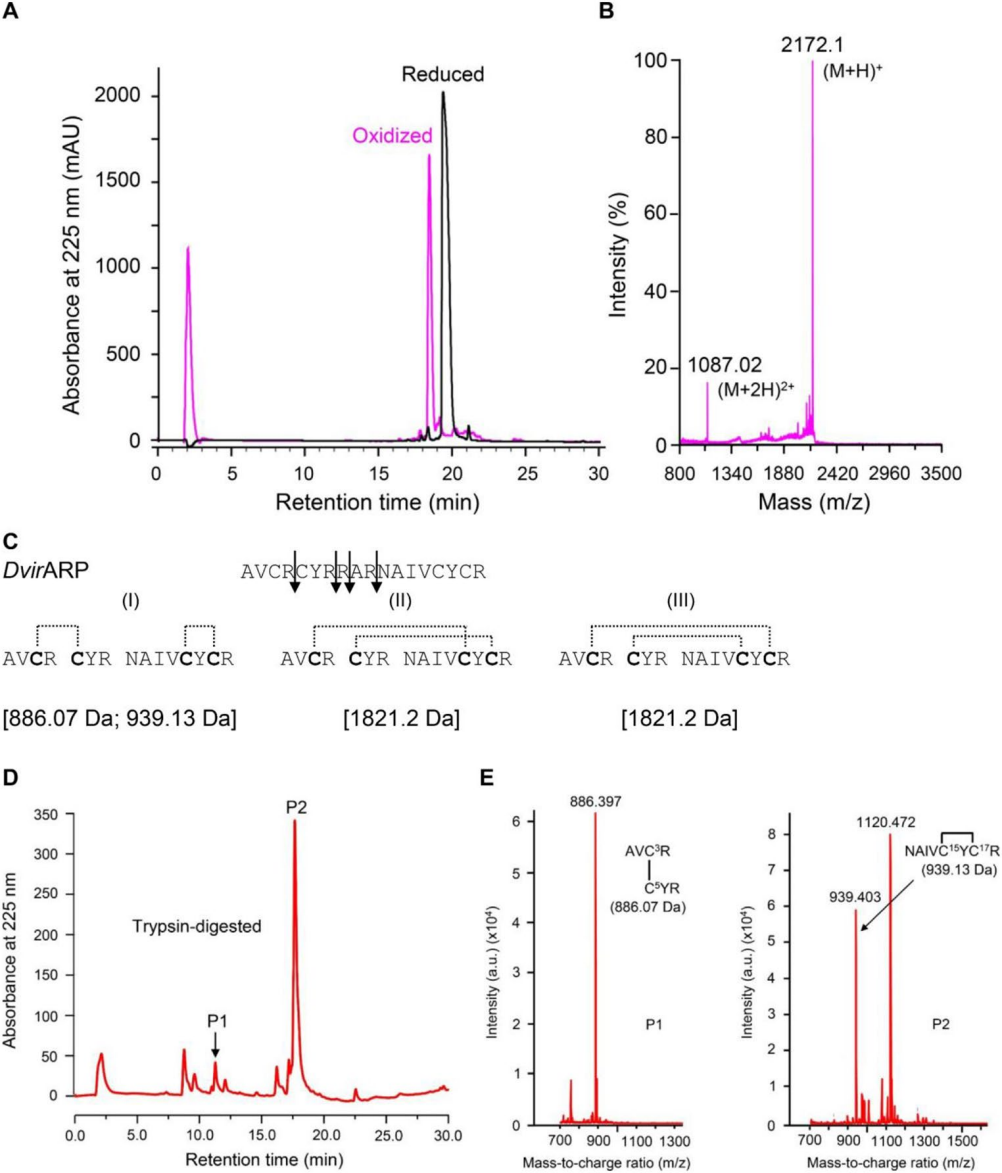
Oxidative refolding and characterization of chemically synthesized *Dvir*ARP. (**A**) RP-HPLC profiles of reduced and oxidized *Dvir*ARP products. The Agilent Zorbax 300SB-C18 (4.6 × 150 mm, 5 μm) was equilibrated with 0.05% TFA in water (v/v) and peptides were eluted from the column with a linear gradient from 0 to 60% acetonitrile in 0.05% TFA within 40 min with a flow rate of 1 mL/min. The UV absorbance was monitored spectrophotometrically at 225 nm. (**B**) MALDI-TOF MS analysis of the oxidized product. Two peaks corresponding to its singly and doubly protonated forms were detected. (**C**–**E**) Determination of disulfide bridges of the oxidized *Dvir*ARP [(**C**) Trypsin digestion sites on *Dvir*ARP and corresponding MWs derived from the digested fragments, with three possible disulfide bridging patterns indicated by dotted lines. (**D**) RP-HPLC profiles of trypsin-digested *Dvir*ARP. P1 and P2, two fragments chosen for MALLDI-TOF analyses. (**E**) Analysis of P1 and P2 by MALDI-TOF MS. Each inset shows the sequences of corresponding fragments with one pair of disulfide bridge and the calculated MWs matching the *m*/*z* values obtained here

To structurally characterize *Dvir*ARP, we chemically synthesized its reduced peptide, from which we produced its folded form via air oxidization in an alkaline environment (Fig. 3A). The oxidized product was purified to homogeneity by RP-HPLC, which was eluted at the retention time (T_*R*_) of 18.5 min, earlier than its reduced form (T_*R*_ of 20 min) (Fig. 3A), indicating that some hydrophobic residues have been buried into the molecular interior and some polar side chains exposed to solvent with folding.

MALDI-TOF MS detected two peaks at m/z = 1087.02 and 2172.1 for the oxidized product, corresponding to its doubly ([M + 2 H]^2+^) and singly ([M + H]^+^) protonated forms, respectively (Fig. 3B). The experimental mass was about 4 Da less than the isotopically averaged MW (2176.6 Da) calculated from the protein sequence, indicating that four hydrogen atoms have been removed from the four cysteines when two disulfide bridges formed during oxidization (Fig. 3B).

To determine the disulfide bonding connectivity pattern in *Dvir*ARP, we employed a method involving trypsin digestion followed by MALDI-TOF MS analysis. This enzyme cleaves specifically peptide bonds at the C-terminal side of lysine or arginine. In theory, four cysteines can form three possible pairing patterns: (1) Cys1–Cys2 and Cys3–Cys4; (2) Cys1–Cys3 and Cys2–Cys4; (**3**) Cys1–Cys4 and Cys2–Cys3 (Fig. 3C). Therefore, based on the information provided by trypsin cleavage and cysteine pairings, we inferred all possible digested fragments (Fig. 3C) which could further be distinguished by MALDI-TOF MS analysis. From the trypsin-digested products, we isolated two fragments by RP-HPLC (namely P1 and P2) (Fig. 3D), from which we identified two peaks at m/z = 886.397 and 939.403 (Fig. 3E) which perfectly matched the theoretical values calculated from the first pairing pattern (886.07 Da and 939.13 Da), indicating that this peptide has a disulfide pattern with Cys1 connected to Cys2 and Cys3 to Cys4.

### *Dvir*ARP exhibits a unique Fold

**Fig. 4:**
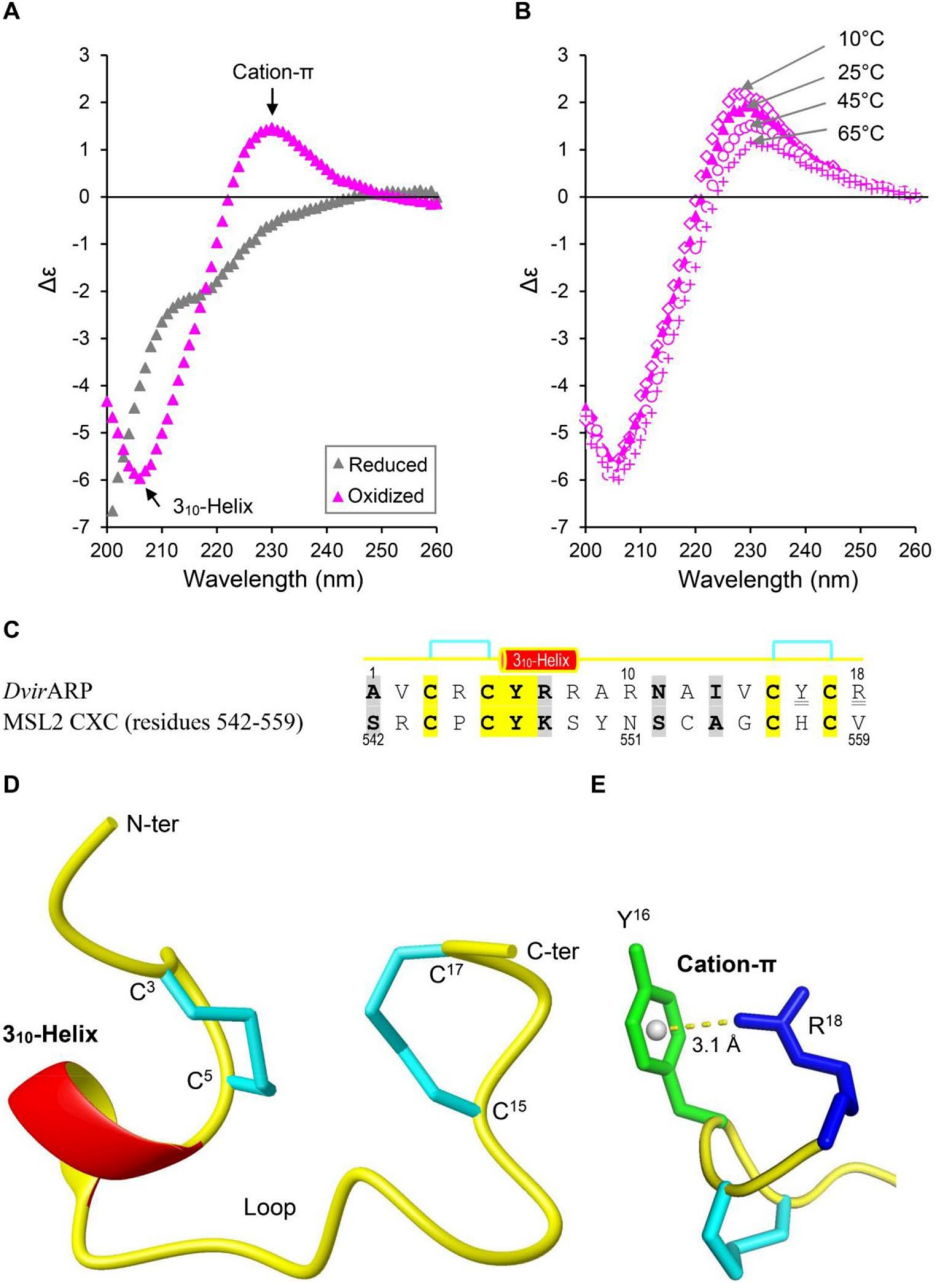
Experiment data-guided structural model of *Dvir*ARP built by comparative modelling. (**A**) Comparison of CD spectra of reduced and oxidized *Dvir*ARP in 5 mM PB buffer (pH7.0). Signature bands for the presence of cation–π and 3_10_-helix are denoted by arrows. (**B**) Thermal denaturation curves of the oxidized product at indicated temperatures. (**C**) Sequence comparison between *Dvir*ARP and the template MSL2 CXC (residues 542–559). Identical and conserved residues are shadowed in *yellow* and *grey*, respectively. Residues involved in cation–π interactions are underlined twice. The predicted secondary structure element and disulfide bridges are extracted from the experiment-based computational model. (**D**) The ribbon model of *Dvir*ARP exhibiting the overall folding, in which disulfide bridge pairings are shown as *cyan* sticks. (**E**) The cation–π interaction on the *Dvir*ARP structure. The highlighted residues are displayed in sticks. The distance between the cationic group (NH_2_) of Arg-18 and the center of the aromatic ring of Tyr-16 is shown

Next, we studied the secondary structure of *Dvir*ARP using CD spectroscopy, an optical spectroscopic technique which can be harnessed to derive the secondary structure information of a protein [47]. As shown in Fig. 4A, the reduced peptide exhibited a disordered, random coil conformation in solution, as identified by a strong negative band before 200 nm (Fig. 4A). In the same solution, its folded peptide showed a structured conformation, as identified by its CD spectra that are dominated by a negative band at 206 nm, an indicator of the presence of a short 3_10_-helix [48], and a positive band around 230 nm, an indicator of the presence of a strong cation–π interaction usually formed between a positively charged amino acid and an aromatic amino acid [49] (Fig. 4A). Variable temperature CD spectra revealed that the signature band at 206 nm overall retained in temperatures ranging from 10 to 65 °C but the signature band around 230 nm decreased with the temperature increase (Fig. 4B), demonstrating that the structure of the helix is more stable than the cation–π interaction.

Filtering with the two structural parameters (3_10_ helix and cation–π interaction) obtained by our CD data, we screened several structural models of *Dvir*ARP created by two representative structural bioinformatics approaches, trRosetta that builds structures of the primary sequence of a protein based on de novo folding, guided by deep learning restraints [50]; and @TOME-2, a web pipeline that allows fold recognition, template selection and MODELLER-based comparative protein structure modelling by satisfaction of spatial restraints [32, 33]. Of the five top models built by trRosetta, no one matched the experimental data in terms of their disulfide pairing patterns and the fold type (Fig. S2; Figs. 3E and 4A). In contrast to trRosetta, @TOME-2 created a model that by and large matched the experimental data (Fig. 4C, D). Through fold recognition, @TOME-2 selected the structure of the MSL2 CXC domain (PDB entry: 4RKH) as template based on the FUGUE sequence-structure homology recognition program. This domain is the C-terminal part of MSL2, a male-specific protein component of the MSL-DCC complex (male-specific lethal dosage compensation complex), which specifically recognizes the MSL recognition element (MRE) sequence motif in the *Drosophila* X chromosome via a single arginine to directly read out dinucleotide sequences from the minor groove of one strand of DNA duplex [34]. *Dvir*ARP shares 50% sequence similarity to the CXC domain, including five identical residues and four conserved residues, particularly they both possess two completely conserved CXC motifs (Fig. 4C). In each motif of MSL2, two cysteines are spatially proximal, which provides a structural basis for the disulfide bridge formation in *Dvir*ARP (Fig. 4D). The model created from the CXC domain contains a short 3_10_ helix spanning residues 6 to 8 (Tyr-Arg-Arg) and a long loop stabilized by two disulfide bridges (Cys1-Cys2 and Cys3-Cys4) (Fig. 4D). Using the CAPTURE program, we detected an energetically significant cation–π interaction between Tyr-16 and Arg-18 with an E_es_ of -3.97 kcal/mol and E_vdw_ of -1.45 kcal/mol

(Fig. 4E). From Fig. 4B, it seemed that this interaction was stable at 25 °C, a suitable temperature for fly survival, suggesting that it could be involved in stabilizing the C-terminus of *Dvir*ARP in normal physiological conditions, in line with the opinion that analogous to hydrophobic effect, hydrogen bond, and ion pair, cation–π interaction also acts as a major force in determining macromolecular structure [51].

### *DvirARP* shows very weak antibacterial activity and is not able to bind DNA

To study the in vitro function of *Dvir*ARP, we firstly evaluated its antimicrobial activity against various microbial strains given its evolutionary relatedness to insect defensins. The strains included 17 Gram-positive bacteria, 22 Gram-negative bacteria, and 9 fungi (Table S3). *Dvir*ARP only exhibited very weak activity against two Gram-positive bacteria (*Bacillus megaterium* and *Micrococcus luteus*) with lethal concentrations (C_*L*_) > 10 µM and no any activity on other bacteria and fungi used here at 1.0 nmol peptide each well when tested in the inhibition zone assay (Table S3). This suggests that this peptide evolved from an insect defensin retains some weak ancestral antibacterial function, a feature previously observed in the origins of animal toxins from proteins of physiological function, which often possess ancestral bioactivities [17, 52].

Next, we analyzed its potential DNA-binding ability considering of its structural similarity to the MSL2 CXC domain (Fig. 4). To this end, we used S15 as test DNA to conduct an electrophoretic mobility shift assay. S15 is the target of the CXC domain of MSL2, which is composed of a 15-bp fragment derived from the MRE motif CES11D1 [34]. We found that this peptide was not able to bind the DNA fragment (Fig. S3A). To account for the result, we created a computational complex of *Dvir*ARP and S15 by a template-based structure modeling of protein-protein interactions [53] from the CXC domain and S15 complex (PDB entry: 4RKH), in which its two arginine legs (Arg-4 and Arg-18) directly insert into the major and minor grooves of S15, respectively (Fig. S3B). Though this appears to be the case of the CXC domain, in which its two arginine legs (Arg-526 and Arg-543) are also involved in DNA recognition [34], two hydrophobic residues (Ala-1 and Val-2) in *Dvir*ARP make a serious steric hindrance that hampers DNA binding (Fig. S3B), which thus provides a structural explanation for the inability of binding DNA for this peptide.

### *DvirARP* confers resistance to Gram-positive bacteria via toxin neutralization

**Fig. 5:**
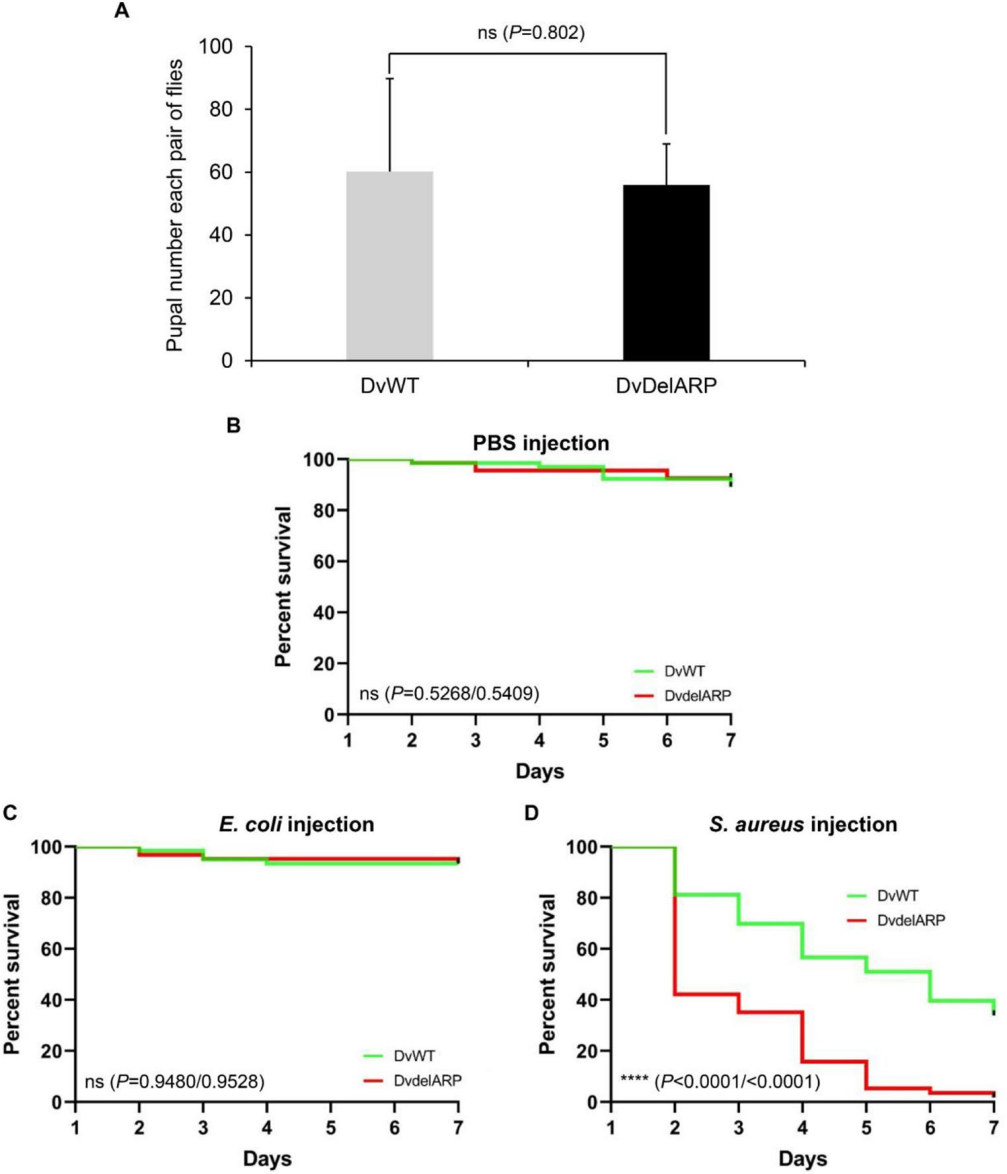
Mutational effects of *Dvir*ARP. (**A**) Comparison of pupation ability of *Dv*WT and *Dv*delARP flies (*n* = 5; ns, no significance). (**B**–**D**) Comparison of survival of *Dv*WT and *Dv*delARP flies to different infections [(**B**) Sterile injury by injecting PBS. (**C**) *E. coli* (OD_600_ = 0.4). (**D**) *S. aureus* (OD_600_ = 0.4)]. Data represent counts from 70 flies per condition. Survival curves were analyzed using Log-rank (Mantel-Cox) test and Gehan-Breslow-Wilcoxon test. ns, no significance and ****P < 0.0001

To explore the in vivo function of *Dvir*ARP, we created a KO mutant of *DvirARP* (named *Dv*delARP) using CRISPR/Cas9 [54] for studying its impact on reproductivity and disease resistance through comparison with the wild-type flies (named *Dv*WT). The frame-shift mutation in *Dv*delARP was verified by PCR and DNA sequencing (Fig. S4). Firstly, we compared the pupal efficiency of each pair of flies (a naive male and a virgin female) (*n* = 5) to analyze the KO effect on the fly reproductivity. The results showed that the KO did not significantly affect the pupal number of flies (*Dv*WT: 60.2 ± 29.6 vs. *Dv*delARP: 56 ± 13) (*P* = 0.804) (Fig. 5A), suggesting that this gene is not involved in regulating reproduction in this fruit fly species. To determine the impact of *Dvir*ARP on injury and host defense, we respectively injected phosphate-buffered saline (PBS, pH 7.5), Gram-negative *E. coli* DH5α and Gram-positive *S. aureus* CGMCC 1.89 cells into female adults of *Dv*WT and *Dv*delARP. The injections with PBS and *E. coli* led to no detectable pathogenicity to *Dv*WT and *Dv*delARP (*P* = 0.5268/0.5409; *P* = 0.9480/0.9528, respectively) (Fig. 5B and C), indicative of its function not implicated in injury and *E. coli* resistance. On the contrary, *S. aureus* showed different degrees of pathogenicity on *Dv*WT and *Dv*delARP but obviously the mutant flies exhibited more susceptibility to the infection than the wild-type flies, as identified by their significantly decreased survival (Fig. 5D) (*P* < 0.0001/<0.0001). This highlights the role of *Dvir*ARP in *D. virilis* host defense against Gram-positive bacterial infection.

**Fig. 6:**
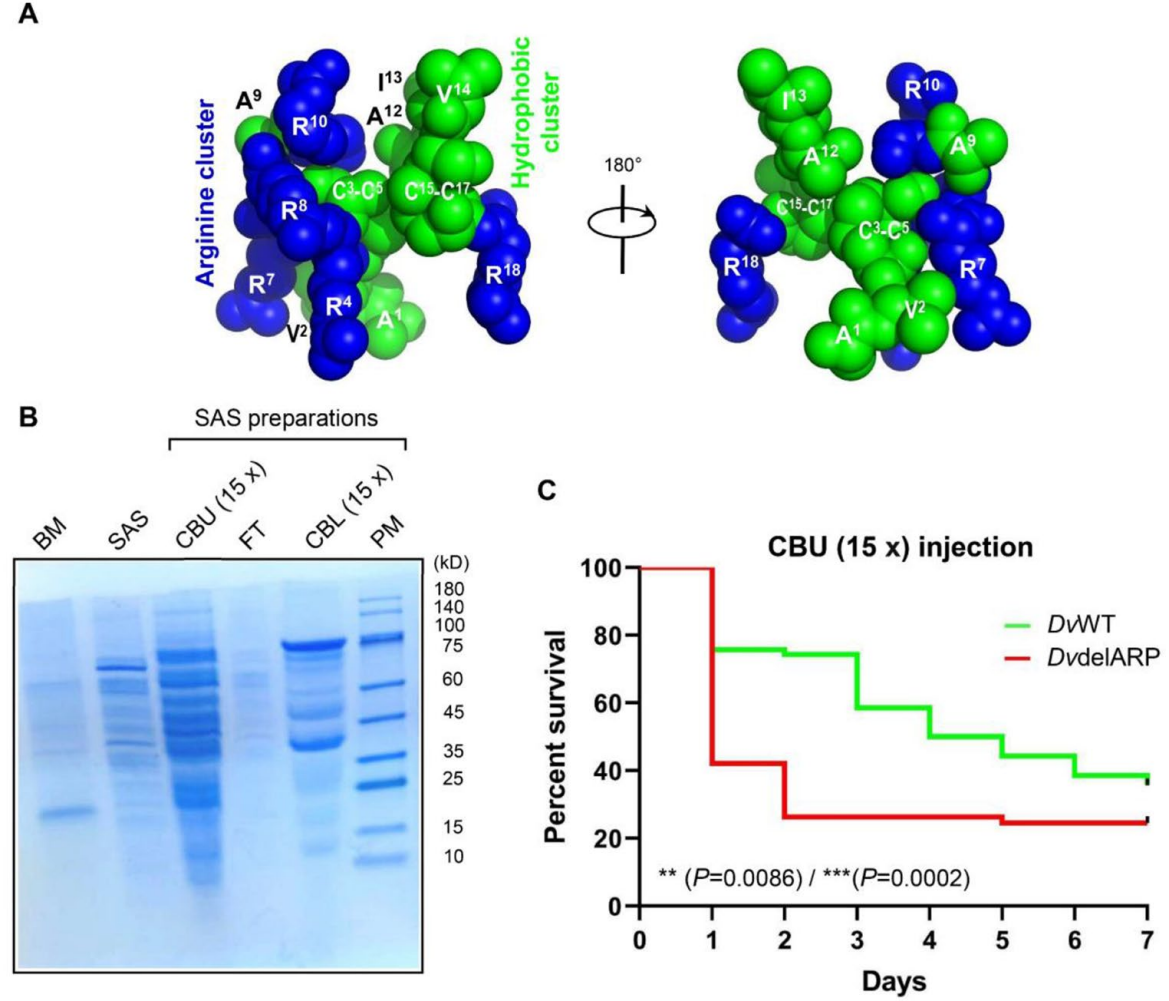
*Dvir*ARP-dependent protection of *D. virillis* flies from the noxious effects of *S. aureus* (SA) toxins. (**A**) *Dvir*ARP adopts an ‘amphipathic’ design. *Blue*, basic (positively charged) amino acids; green, hydrophobic (‘oily’) amino acids. Other amino acids are not shown for clarification. (**B**) SDS-PAGE profiles of SA supernatant preparations. BM, Broth medium; SAS, *S. aureus* supernatant; CBU (15x): concentration by ultrafiltration (15x); FT: flow-through; CBL (15 x): concentration by lyophilization (15x); PM: protein marker. (**C**) Comparison of survival of *Dv*delARP and *Dv*WT flies to SAS [CBU(15x)]. Data represent counts from 70 flies per condition. Survival curves were analyzed using Log-rank (Mantel-Cox) test and Gehan-Breslow-Wilcoxon test. **P < 0.01 and ***P < 0.001

To study whether this role is a consequence of direct antibacterial function of *Dvir*ARP within insects, we mimicked the environment in inhibition zone assay against *S. aureus* CGMCC 1.89 through dissolving the peptide in insect saline or the haemolymph serum collected from *Dv*WT or *Dv*delARP female adults. The results showed that in all these conditions this peptide exhibited no detectable antibacterial activity on this strain (Fig. S5). The lack of a direct bactericidal activity on *S. aureus* (Table S3; Fig. S5) implies that the observed protective role of *Dvir*ARP should not be due to a resistance-medicated mechanism other than more likely a consequence of toxin neutralization, which is supported by the fact that *S. aureus* secretes a variaty of toxins and extracellular enzymes to destroy the host’s cells [55] and the proposal that amphipathic proteins without antimicrobial properties likely work primarily as toxin-destablizing innate defense factors [56]. Structurally, *Dvir*ARP adopts an ‘amphipathic’ design [57], in which four clustered positively charged arginine and multiple clustered hydrophobic amino acids are spatially separated on two sides of the molecule (Fig. 6A). To verify our speculation, we injected the *S. aureus* culture supernatant into female flies, which contained multiple protein components with different molecular sizes (Fig. 6B). We found that the toxin-containing supernatant led to a lower survival rate in the mutant flies than the wild-type flies (*P* = 0.0086/*P* = 0.0002) (Fig. 6C), indicating that *Dvir*ARP indeed works as an antitoxin factor to counteract *S. aureus*-secreted toxins and thereby improve the fly survial rate.

### *Dvir*ARP-related peptides restrictedly distributed in species of *Drosophila* subgenus

To study the phylogenetic distribution of *Dvir*ARP to trace its origin, we conducted a systematic TBLASTN search of the *Drosophila* genome database. This led to the discovery of orthologs of *Dvir*ARP derived from other five *Drosophila* species, including *D. albomicans*, *D. nasuta*, *D. grimshawi*, *D. americana*, and *D. montana* (Fig. S6). Similar to *DvirARP*, all these orthologous genes encode a precursor comprising of a signal peptide, a propeptide and a mature peptide, with detectable similarity to their paralogous defensins. For example, in the mature region *Dnas*ARP and *Dalb*ARP share nearly completely identical C-terminal sequence with many defensins, all terminated by Val-Cys-Val-Cys-Arg-Arg (Fig. S6; also see Fig. 1B). In particular, sequence similarity further extends to their precursor processing signals, including the cleavage of signal peptides mostly at an alanine and the removal of propeptides by a Kex2-like processing endoprotease recognizing a common cleavage motif (RXKR; X, any amino acid) (Fig. S6). The mature peptides are composed of 17 to 19 residues, shorter in size than their defensin paralogos (40 residues). With the exception of the deletion mutation mentioned previously, point mutations occurred to accumulate high content of basic residues (22–42%) without acidic residues. In contrast to the ARPs, the *Drosophila* defensins contained only 10–18% basic residues but 2.5–7.5% acidic residues (Fig. S6).

**Fig. 7:**
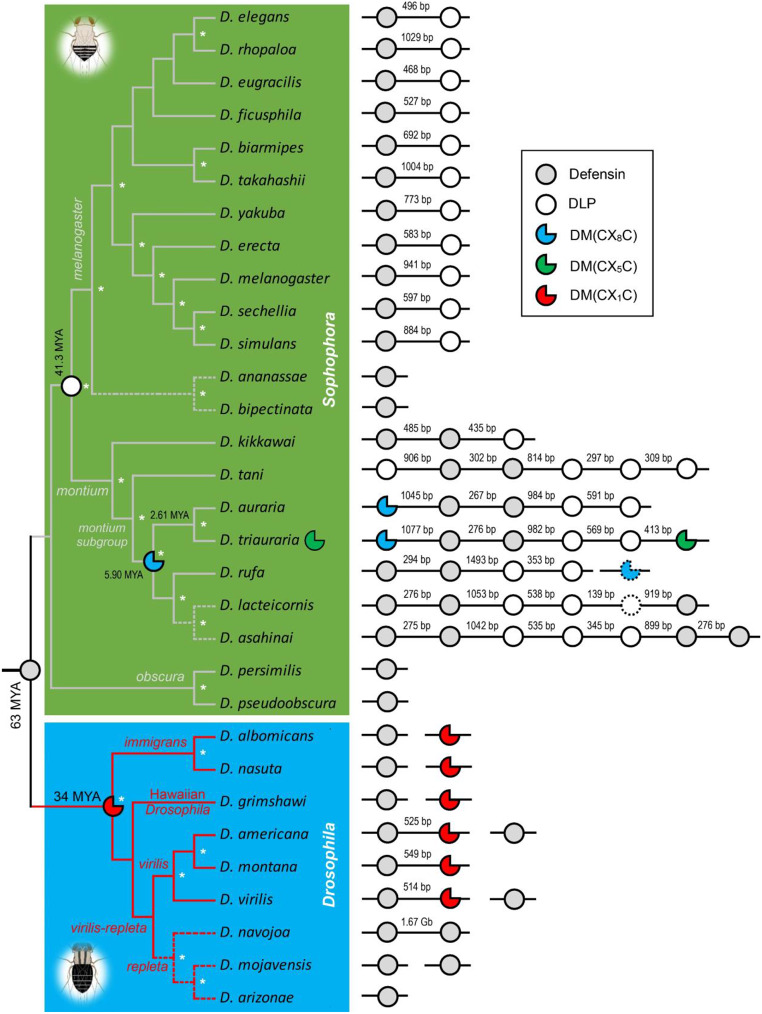
The evolutionary relationships of the genus *Drosophila* for displaying the distribution of insect defensins and evolutionarily related deletion mutants. This tree elucidated by the *RP49* gene identifies two distinct branches that well correspond to two *Drosophila* subgenus (*Sophophora* and *Drosophila*). Asterisks shown at nodes indicate the bootstrap test (500 replicates) confidence levels ≥ 50%. Species groups are shown here. The branches with gene lost are denoted by dotted lines. The origins of defensins and the deletion mutants are denoted by different symbols with different colors. The gene clusters on the chromosomes are shown at the right of the tree. Pseudogenes are represented by dotted symbols. Divergence times for the *montium* subgroup were estimated via http://www.timetree.org/ and other divergence times were cited from literatures [58, 59]. MYA, million years ago; DLP, defensin-like peptide; DM, deletion mutation

The ARPs [i.e. DM(CX_1_C)] are encoded by single copy genes in the species belonging to the *Drosophila* subgenus with a history of 34 MYA (Fig. 7). These peptides are restrictedly distributed in three species groups of the subgenus, including the *virilis* group (*D. americana*, *D. montana* and *D. virilis*), the *immigrans* group (*D. albomicans* and *D. nasuta*) and the Hawaiian Drosophila *D. grimshawi*. Of them, three species were identified to have a defensin–ARP gene cluster with a distance of ~ 500 bp–1.6 Gb (Fig. 7). Three species in this subgenus (*D. navojoa*, *D. mojavensis* and *D. arizonae*) contained no ARP gene, which all belong to the monophyletic *repleta* species group as seen in the *Drosophila* tree built based on the *RP49* gene sequence (Fig. 7). We thus speculated that gene loss occurred once in the common ancestor of the *repleta* species group (Fig. 7).

### Independent deletion variations of defensins between two *Drosophila* subgenus

To study whether the defensin deletion mutation event also occurs in the Sophophora subgenus, we conducted new TBLASTN searches of the *Drosophila* genome database with insect defensins as queries. Besides the hits of new insect defensins (Fig. S7), we found three new classes of defensin variants originated from deletion mutations in this subgenus: (1) Defensin-like peptides (DLPs) with the N-terminal loop deletion (Fig. S8). Such deletions did not alter the fold due to the conservation of the six cysterine residues for folding (Fig. S9). Some DLPs developed one single free cysteine, which could be used to form an interchain disulfide bridge (Fig. S10), as previously observed in some bacterial defensins [60]. The history of DLPs could be traced back to the common ancestor of the *melanogaster*-*motium* clade with a history of 41.3 MYA (Fig. 7); (2) Deletion mutants [DM(CX_8_C)] in the *montium* subgroup with a history of 5.90 MYA (Fig. 7; Fig. S11). These peptides are natural variants of defensins with the region covering the second and third cyeteine residues (C2 and C3) deleted. There are eight residues between the first two cysteines, thus termed CX_8_C; (3) Deletion mutants [DM(CX_5_C)] in *D. triauraria* that diverged from *D. triauraria* 2.61 MYA via a three-residues longer deletion in defensins relative to the DM(CX_8_C) peptides (Fig. 7; Fig. S11; Fig. S12). In the latter two cases, due to the deletion of two crucial cysteine residues for the formation of a defensin fold, their fold types have been altered via disulfide bridge reorganization (Fig. S12). The observation that genetic deletions in insect defensins independently occurred between the two subgenus of *Drosophila* provides new support for the evolvability of this class of immune effectors in *Drosophila*. Further studies of the deletion variants in the *montium* subgroup [61] will lead to new findings in terms of their structures and biological functions, which will help explore the significance of parallel deletion events between *Drosophila* subgenus.

## Discussion

**Fig. 8:**
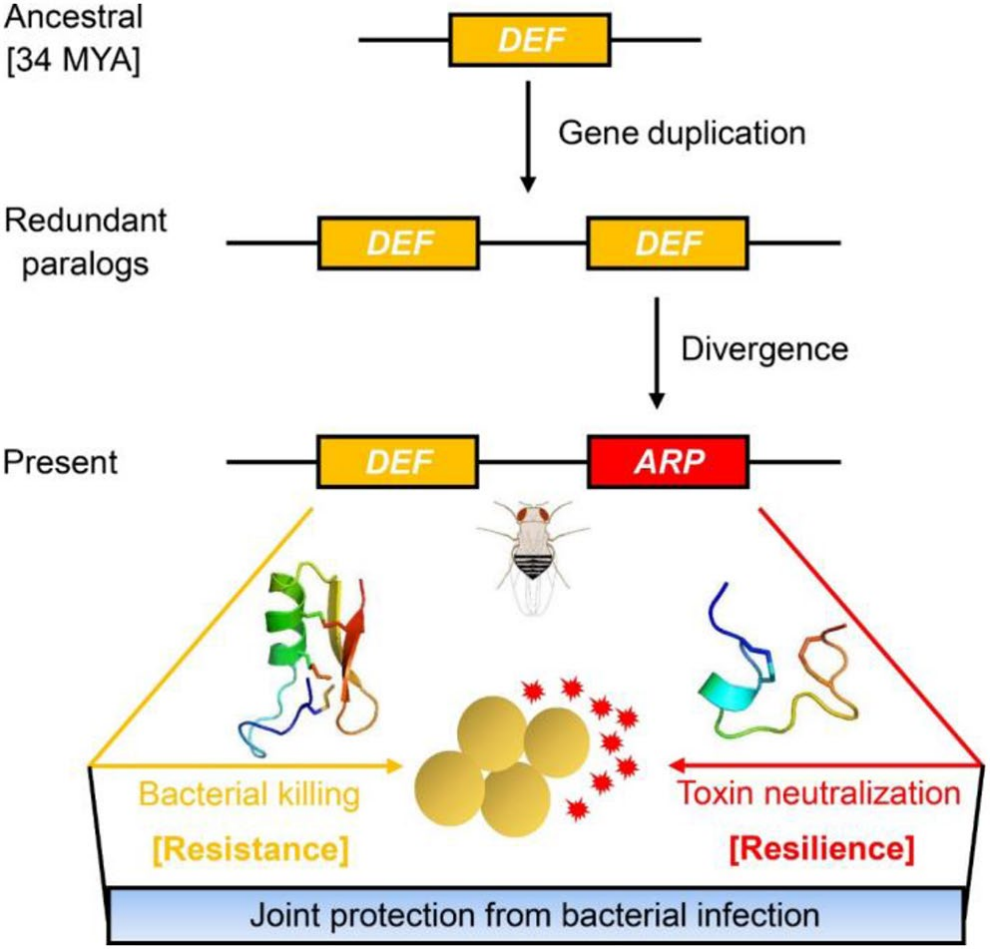
Evolution of the novel antitoxin activity in ARPs of the *Drosophila* subgenus from an ancestral defensin gene (*DEF*) following gene duplication. Golden ovals represent *S. aureus* cells and *red* explosion shapes represent their secreted toxins. The structures presented here are *Dvir*DEF (*left*) and *Dvir*ARP (*right*) models, respectively

It is long known that new genes typically originate as products of duplications and of the vast majority of gene duplicates, only a few survivors evolve new functions [62]. In this study, we report the discovery of a lineage-specific gene duplication-mediated new gene origination in the *Drosophila* subgenus, including the mechanism that generates a new fold to the role of the new gene in host defense (Fig. 8). This gene was created by genetic deletion of partial sequence of an ancestral defensin following gene duplication after divergence of the *Drosophila* subgenus from the Sophophora subgenus. We demonstrate that a dramatic fold change can occur in evolution, which is in contrast to the generally accepted opinion that protein spatial structures are more conserved in evolution than primary sequences [63, 64]. In our example, a three-disulfide-linked defensin fold is changed to a two-disulfide-linked small helix-loop structure. Such alteration appears to be related to the genetic deletion of a specific region in a defensin fold, which contains two conserved cysteine residues (Cys2 and Cys3), leading to the replacement of the initial Cys2–Cys5 and Cys3–Cys6-linked disulfide bridges by a reorganized Cys5–Cys6-linked disulfide bridge, accompanying the reservation of the Cys1–Cys4-linked disulfide bridge (Note: Cys numbered according to their position in the defensin) (Fig. S6). Intriguingly, deletion of the equivalent region in a contemporary insect defensin produced a β-sheet structure with two reorganized disulfide bridges (Cys1–Cys6 and Cys4–Cys5) [65]. This structure is analogous to its native state in the parent peptide but remarkably different from that of *Dvir*ARP, suggesting that the evolution of a new fold from a progenitor insect defensin is a gradual process and the β-sheet structure could represent an evolutionary intermediate state. This suggestion is also highly consistent with the opinion that evolution is often gradual [66]. Deletion-mediated cysteine loss followed by disulfide bridge reorganization is also found in the evolution of mammalian θ-defensins which are produced by binary ligation of two truncated α-defensins, resulting in fold change and functional diversification [67]. Our finding is also in line with the opinion that insertion and deletion (Indel) trigger dramatic structural transitions in evolution [68] but remarkably different from the fold change in the evolution of homologous fungal defensin-like peptides, which is achieved by motif change other than cysteine deletion in evolution [69], and also different from the finding that protein structures could be changed during the process of evolution by just few mutations in sequences [70, 71].

Since *Dvir*ARP and its orthologous sequences show a difference from insect defensin sequences in their residue contents, as identified by their more basic residues with no acidic residues (Fig. S6), we infer that in this new fold, continuous point mutations to introduce more basic amino acids could help assemble a novel amphipathic architecture. This would further drive their functional diversification from a bactericidal defensin to an antitoxin peptide to counteract bacteria producing toxins. An increase in the arginine content leading to new biochemical properties and functional change is also observed previously in the evolution of the gene for eosinophil cationic protein (ECP) [72]. Since in eukaryotic genomes indel mutations often induce an increase in the substitution rate of their flanking regions [73, 74], we can infer that the evolutionary change observed in *Dvir*ARP could occur largely by deletion-driven new point mutations changing the peptide’s biochemical properties, thus giving rise to functional innovation. This inference is also consistent with the finding of the loop-region deletion inducing an increase in the substitution rate of their flanking regions mediating the extensive sequence divergence between defensins and scorpion toxins [17] and the insertion-elicited accelerated substitutions in a specific flanking loop mediating the emergence of ACE2 binding trait in SARS-CoV-2 Spike receptor-binding domain (RBD) [75]. It is known that multiple human defensins (e.g. HNP-1, HD5, hBD2 and Retrocyclins) have been identified to have ability of unfolding of bacterial toxins via their amphipathic design [76–82] and even some amphipathic proteins without antimicrobial properties might act primarily as toxin-destablizing innate defense factors [56]. Obviously, *Dvir*ARP belongs to the latter case, in which the peptide could be attached to the negatively charged regions of bacterial toxins via its arginine cluster-mediated electrostatic interactions and its hydrophobic region could interact with the exposed hydrophobic surface of a partially unfolded state of the toxins, as proposed for the mechanism of human defensins in unfolding of thermodynamically unstable regions of bacterial protein toxins [77].

Meanwhile, our study highlights the evolution of the *DvirARP* gene at two levels, a concept initially proposed in the evolution of genes between humans and chimpanzees [83, 84]: changes in the coding region as discussed above and changes in the regulatory region, in which minor changes in two regulatory motifs (κB motif and Inr) lead to changes in the regulation of gene expression. The latter case is similar to many evolving genes that changes their expression through changes in sites that bind transcription factors (TFs). While it is still unclear the output of a constitutive gene expression pattern in *Dvir*ARP, this is obviously different from the previously reported antitoxin peptides in *Drosophila* where their expression is often induced by microbial infections, but similar to some human antitoxin defensins (e.g. α-defensins) that also are constitutively expressed in human oral cavity [60]. In addition, the constitutive manner could hit the biological functions of this gene beyond antitoxin activity. In particular, if we consider that arginine-rich peptides have two remarkable functional features: (1) They often present multiple interfaces for RNA binding [85]; (2) Arginine-rich peptides are a representative class of cell-penetrating peptides [86]. Thus, the possibility of *Dvir*ARP and its orthologs as a regulator to control cellular physiology via cellular uptake to bind intracellular targets (e.g. RNAs) is not excluded.

According to the opinion that gene loss can sometimes be beneficial [87], the loss of the *Dvir*ARP orthologs in the *repleta* species group could thus suggest that the evolution of an enhanced resilience may be not beneficial to all *Drosophila* species given the diversity in their survival environments. For instance, *D. melanogaster* breeds on fruits fermented by *Saccharomyces cerevisia*e, whereas *D. virilis* breeds on slime flux and decaying bark of tree housing a variety of bacteria, yeasts, and molds [88], which could drive extensive differentiation of their innate immune system during evolution [89].

In summary, this study further extends the genetic deletion-mediated evolvability scope of insect defensins from fold preservation (i.e. origins of scorpion toxins [17] and *Drosophila* DLPs described here) to fold alterations (i.e. origins of DM(CX_1_C) to DM(CX_8_C)). It represents a new advance in the study of new gene origination, in which an old gene evolved a new fold to enable it to bind bacterial toxins rather than bacteria themselves. This adds one dimension in host defense relative to its ancestral gene. Further studies to clarify the toxin targets of *Dvir*ARP and the molecular basis of toxin neutralization will provide new insights into the resilience mechanism and the relationship with disease resistance in *D. virilis* and related species as well as guidance for design of antitoxin drugs against *S. aureus* infection. Finally, the discovery of the diversity of insect defensin evolvability is also important, not only for understanding natural evolution of host defense but also for peptide engineering and design.

### Electronic supplementary material

Below is the link to the electronic supplementary material.

Supplementary Material 1

Supplementary Material 2

## Data Availability

cDNA sequences encoding *Dvir*DEF and *Dvir*ARP and their genomic DNA sequence have been deposited in the GenBank database (https://ncbi.nlm.nih.gov/) under accession number of KF971894, KF971895 and KF971893. All other data are available in the main text or the Supporting Information. Correspondence and requests for materials should be addressed to S. Z.

## Abbreviations

3D: Three-dimensional
AMP: Antimicrobial peptide
ARP: Arginine-rich peptide
CD: Circular dichroism
CDS: Coding DNA sequence
CHCA: α-Cyano-4-hydroxycinnamic acid
CITD: Classical insect-type defensin
C_*L*_: Lethal concentration
CSαβ: Cysteine-stabilized α-helix and β-sheet
DEPC: Diethyl pyrocarbonate
DLP: Defensin-like peptide
DPE: Downstream core promoter element
dsRNA: Double-stranded RNA
ECP: Eosinophil cationic protein
Imd: Immune deficiency
Indel: Insertion and deletion
Inr: Initiator
KO: Knockout
KTx: K^+^-channel-targeted neurotoxin
MALDI-TOF MS: Matrix-assisted laser desorption/ionization time of flight mass spectrometry
MOE: Molecular operating environment
MPAC: Modified pro-domain of attacin C
MRE: MSL recognition element
MSL: Male-specific lethal
MW: Molecular weight
MYA: Million years ago
RBD: Receptor-binding domain
RP-HPLC: Reversed-phase high-performance liquid chromatography
SDS-PAGE: Sodium dodecyl sulfate polyacrylamide gel electrophoresis
TF: Transcription factor
TFBS: Transcription factor binding site

## References

[1] Lavine MD, Strand MR (2002). Insect hemocytes and their role in immunity. Insect Biochem Mol Biol.

[2] Lemaitre B, Hoffmann J (2007). The host defense of *Drosophila melanogaster*. Annu Rev Immunol.

[3] Marmaras VJ, Lampropoulou M (2009). Regulators and signalling in insect haemocyte immunity. Cell Signal.

[4] Imler JL, Bulet P (2005). Antimicrobial peptides in *Drosophila*: structures, activities and gene regulation. Chem Immunol Allergy.

[5] Hanson MA, Dostálová A, Ceroni C, Poidevin M, Kondo S, Lemaitre B (2019). Synergy and remarkable specificity of antimicrobial peptides *in vivo* using a systematic knockout approach. Elife.

[6] Huang J, Lou Y, Liu J, Bulet P, Cai C, Ma K, Jiao R, Hoffmann JA, Liégeois S, Li Z, Ferrandon D (2023). A toll pathway effector protects *Drosophila* specifically from distinct toxins secreted by a fungus or a bacterium. Proc Natl Acad Sci U S A.

[7] Xu R, Lou Y, Tidu A, Bulet P, Heinekamp T, Martin F, Brakhage A, Li Z, Liégeois S, Ferrandon D (2023). The toll pathway mediates *Drosophila* resilience to *aspergillus* mycotoxins through specific Bomanins. EMBO Rep.

[8] Clemmons AW, Lindsay SA, Wasserman SA (2015). An effector peptide family required for *Drosophila* toll-mediated immunity. PLoS Pathog.

[9] Lindsay SA, Lin SJH, Wasserman SA (2018). Short-form bomanins mediate humoral immunity in *Drosophila*. J Innate Immun.

[10] Hanson MA, Cohen LB, Marra A, Iatsenko I, Wasserman SA, Lemaitre B (2021). The *Drosophila* Baramicin polypeptide gene protects against fungal infection. PLoS Pathog.

[11] Dimarcq JL, Hoffmann D, Meister M, Bulet P, Lanot R, Reichhart JM, Hoffmann JA (1994). Characterization and transcriptional profiles of a *Drosophila* gene encoding an insect defensin. A study in insect immunity. Eur J Biochem.

[12] Koehbach J (2017). Structure-activity relationships of insect defensins. Front Chem.

[13] Hanzawa H, Shimada I, Kuzuhara T, Komano H, Kohda D, Inagaki F, Natori S, Arata Y (1990). 1H nuclear magnetic resonance study of the solution conformation of an antibacterial protein, sapecin. FEBS Lett.

[14] Cornet B, Bonmatin JM, Hetru C, Hoffmann JA, Ptak M, Vovelle F (1995). Refined three-dimensional solution structure of insect defensin A. Structure.

[15] Zhu S, Gao B, Tytgat J (2005). Phylogenetic distribution, functional epitopes and evolution of the CSalphabeta superfamily. Cell Mol Life Sci.

[16] Zhu S (2008). Discovery of six families of fungal defensin-like peptides provides insights into origin and evolution of the CSαβ defensins. Mol Immunol.

[17] Zhu S, Peigneur S, Gao B, Umetsu Y, Ohki S, Jan T (2014). Experimental conversion of a defensin into a neurotoxin: implications for origin of toxic function. Mol Biol Evol.

[18] Manniello MD, Moretta A, Salvia R, Scieuzo C, Lucchetti D, Vogel H, Sgambato A, Falabella P (2021). Insect antimicrobial peptides: potential weapons to counteract the antibiotic resistance. Cell Mol Life Sci.

[19] Takeuchi K, Takahashi H, Sugai M, Iwai H, Kohno T, Sekimizu K, Natori S, Shimada I (2004). Channel-forming membrane permeabilization by an antibacterial protein, sapecin: determination of membrane-buried and oligomerization surfaces by NMR. J Biol Chem.

[20] Blandin S, Moita LF, Köcher T, Wilm M, Kafatos FC, Levashina EA (2002). Reverse genetics in the mosquito Anopheles gambiae: targeted disruption of the defensin gene. EMBO Rep.

[21] Caspermeyer J (2014). How a scorpion gets its sting. Mol Biol Evol.

[22] Payne JL, Wagner A (2019). The causes of evolvability and their evolution. Nat Rev Genet.

[23] Buda K, Miton CM, Fan XC, Tokuriki N (2023). Molecular determinants of protein evolvability. Trends Biochem Sci.

[24] Zhu S, Gao B (2006). Molecular characterization of a new scorpion venom lipolysis activating peptide: evidence for disulfide bridge-mediated functional switch of peptides. FEBS Lett.

[25] McPherson MJ, Moller SG (2000). PCR.

[26] Engström Y, Kadalayil L, Sun SC, Samakovlis C, Hultmark D, Faye I (1993). Kappa B-like motifs regulate the induction of immune genes in *Drosophila*. J Mol Biol.

[27] Petersen UM, Kadalayil L, Rehorn KP, Hoshizaki DK, Reuter R, Engström Y (1999). Serpent regulates *Drosophila* immunity genes in the larval fat body through an essential GATA motif. EMBO J.

[28] Kadonaga JT (2012). Perspectives on the RNA polymerase II core promoter. Wiley Interdiscip Rev Dev Biol.

[29] Burke TW, Kadonaga JT (1997). The downstream core promoter element, DPE, is conserved from *Drosophila* to humans and is recognized by TAFII60 of *Drosophila*. Genes Dev.

[30] Haberle V, Stark A (2018). Eukaryotic core promoters and the functional basis of transcription initiation. Nat Rev Mol Cell Biol.

[31] Shi J, Blundell TL, Mizuguchi K (2001). FUGUE: sequence-structure homology recognition using environment-specific substitution tables and structure-dependent gap penalties. J Mol Biol.

[32] Pons JL, Labesse G (2009) @TOME-2: a new pipeline for comparative modeling of protein-ligand complexes. Nucleic Acids Res 37(Web Serv Issue W485–491. 10.1093/nar/gkp368 10.1093/nar/gkp368 PMC2703933 19443448

[33] Webb B, Sali A (2016) Comparative protein structure modeling using MODELLER. Curr Protoc Bioinf 54. 5.6.1–5.6.37 10.1002/cpbi.3 PMC5031415 27322406

[34] Zheng S, Villa R, Wang J, Feng Y, Wang J, Becker PB, Ye K (2014). Structural basis of X chromosome DNA recognition by the MSL2 CXC domain during *Drosophila* dosage compensation. Genes Dev.

[35] Benkert P, Tosatto SC, Schomburg D (2008). QMEAN: a comprehensive scoring function for model quality assessment. Proteins.

[36] Gallivan JP, Dougherty DA (1999). Cation-pi interactions in structural biology. Proc Natl Acad Sci U S A.

[37] Tamura K, Stecher G, Kumar S (2021). MEGA11: Molecular Evolutionary Genetics Analysis Version 11. Mol Biol Evol.

[38] Zhu S, Gao B (2017). Positive selection in cathelicidin host defense peptides: adaptation to exogenous pathogens or endogenous receptors?. Heredity (Edinb).

[39] Ekengren S, Hultmark D (1999). *Drosophila* cecropin as an antifungal agent. Insect Biochem Mol Biol.

[40] Gratz SJ, Ukken FP, Rubinstein CD, Thiede G, Donohue LK, Cummings AM, Oconnor-Giles KM (2014). Highly specific and efficient CRISPR/Cas9-catalyzed homology-directed repair in *Drosophila*. Genetics.

[41] Ashburner M, Roote J, Sullivan (2000). Laboratory Culture of *Drosophila*, Chap. 35. Drosophila protocols.

[42] Rockwell NC, Krysan DJ, Komiyama T, Fuller RS (2002). Precursor processing by kex2/furin proteases. Chem Rev.

[43] Cociancich S, Ghazi A, Hetru C, Hoffmann JA, Letellier L (1993). Insect defensin, an inducible antibacterial peptide, forms voltage-dependent channels in *Micrococcus luteus*. J Biol Chem.

[44] Kadalayil L, Petersen UM, Engström Y (1997). Adjacent GATA and kappa B-like motifs regulate the expression of a *Drosophila* immune gene. Nucleic Acids Res.

[45] Engström Y (1999). Induction and regulation of antimicrobial peptides in *Drosophila*. Dev Comp Immunol.

[46] Georgakopoulos-Soares I, Deng C, Agarwal V, Chan CSY, Zhao J, Inoue F, Ahituv N (2023). Transcription factor binding site orientation and order are major drivers of gene regulatory activity. Nat Commun.

[47] Miles AJ, Janes RW, Wallace BA (2021). Tools and methods for circular dichroism spectroscopy of proteins: a tutorial review. Chem Soc Rev.

[48] Toniolo C, Polese A, Formaggio F, Crisma M, Kamphuis J (1996). Circular dichroism spectrum of a peptide 3_10_-Helix. J Am Chem Soc.

[49] Peter B, Polyansky AA, Fanucchi S, Dirr HW (2014). A lys-trp cation–π interaction mediates the dimerization and function of the chloride intracellular channel protein 1 transmembrane domain. Biochemistry.

[50] Du Z, Su H, Wang W, Ye L, Wei H, Peng Z, Anishchenko I, Baker D, Yang J (2021). The trRosetta server for fast and accurate protein structure prediction. Nat Protoc.

[51] Dougherty DA (2013). The cation–π interaction. Acc Chem Res.

[52] Fry BG (2005). From genome to venome: molecular origin and evolution of the snake venom proteome inferred from phylogenetic analysis of toxin sequences and related body proteins. Genome Res.

[53] Szilagyi A, Zhang Y (2014). Template-based structure modeling of protein-protein interactions. Curr Opin Struct Biol.

[54] Bassett AR, Tibbit C, Ponting CP, Liu JL (2014). Highly efficient targeted mutagenesis of *Drosophila* with the CRISPR/Cas9 system. Cell Rep.

[55] Tam K, Torres VJ (2019) *Staphylococcus aureus* secreted toxins and extracellular enzymes. Microbiol Spectr 7:10.1128/microbiolspec.GPP3-0039-2018 2018. 10.1128/microbiolspec.gpp3-0039-2018 PMC6422052 30873936

[56] Schröder JM (2014). Revealing the Achilles heel of bacterial toxins. Immunity.

[57] Zasloff M (2002). Antimicrobial peptides of multicellular organisms. Nature.

[58] Tamura K, Subramanian S, Kumar S (2004). Temporal patterns of fruit fly (*Drosophila*) evolution revealed by mutation clocks. Mol Biol Evol.

[59] Suvorov A, Kim BY, Wang J, Armstrong EE, Peede D, D’Agostino ERR, Price DK, Waddell P, Lang M, Courtier-Orgogozo V, David JR, Petrov D, Matute DR, Schrider DR, Comeault AA (2022). Widespread introgression across a phylogeny of 155 *Drosophila* genomes. Curr Biol.

[60] Zhu S, Gao B, Umetsu Y, Peigneur S, Li P, Ohki S, Tytgat J (2022). Adaptively evolved human oral actinomyces-sourced defensins show therapeutic potential. EMBO Mol Med.

[61] Conner WR, Delaney EK, Bronski MJ, Ginsberg PS, Wheeler TB, Richardson KM, Peckenpaugh B, Kim KJ, Watada M, Hoffmann AA, Eisen MB, Kopp A, Cooper BS, Turelli M (2021). A phylogeny for the *Drosophila* montium species group: a model clade for comparative analyses. Mol Phylogenet Evol.

[62] Lynch M, Conery JS (2000). The evolutionary fate and consequences of duplicate genes. Science.

[63] Holm L, Sander C (1996). Mapping the protein universe. Science.

[64] Holm L, Sander C (1997). New structure–novel fold?. Structure.

[65] Gao B, Li P, Zhu S (2024). Single deletion unmasks hidden anti-gram-negative bacterial activity of an insect defensin-derived peptide. J Med Chem.

[66] Futuyma DJ, Kirkpatrick M (2017) Evolution, 4th edn. Sinauer Associates

[67] Tang YQ, Yuan J, Osapay G, Osapay K, Tran D, Miller CJ, Ouellette AJ, Selsted ME (1999). A cyclic antimicrobial peptide produced in primate leukocytes by the ligation of two truncated alpha-defensins. Science.

[68] Tóth-Petróczy A, Tawfik DS (2014). Hopeful (protein InDel) monsters?. Structure.

[69] Wu Y, Gao B, Zhu S (2017). New fungal defensin-like peptides provide evidence for fold change of proteins in evolution. Biosci Rep.

[70] Cordes MH, Walsh NP, McKnight CJ, Sauer RT (1999). Evolution of a protein fold *in vitro*. Science.

[71] Meier S, Jensen PR, David CN, Chapman J, Holstein TW, Grzesiek S, Ozbek S (2007). Continuous molecular evolution of protein-domain structures by single amino acid changes. Curr Biol.

[72] Zhang J, Rosenberg HF, Nei M (1998). Positive darwinian selection after gene duplication in primate ribonuclease genes. Proc Natl Acad Sci U S A.

[73] Tian D, Wang Q, Zhang P, Araki H, Yang S, Kreitman M, Nagylaki T, Hudson R, Bergelson J, Chen J (2008). Single-nucleotide mutation rate increases close to insertions/deletions in eukaryotes. Nature.

[74] Zhang Z, Huang J, Wang Z, Wang L, Gao P (2011). Impact of indels on the flanking regions in structural domains. Mol Biol Evol.

[75] Gao B, Zhu S (2023). Mutation-driven parallel evolution in emergence of ACE2-utilizing sarbecoviruses. Front Microbiol.

[76] Kim C, Gajendran N, Mittrücker HW, Weiwad M, Song YH, Hurwitz R, Wilmanns M, Fischer G, Kaufmann SH (2005). Human alpha-defensins neutralize anthrax lethal toxin and protect against its fatal consequences. Proc Natl Acad Sci U S A.

[77] Kudryashova E, Quintyn R, Seveau S, Lu W, Wysocki VH, Kudryashov DS (2014). Human defensins facilitate local unfolding of thermodynamically unstable regions of bacterial protein toxins. Immunity.

[78] Kudryashova E, Seveau S, Lu W, Kudryashov DS (2015). Retrocyclins neutralize bacterial toxins by potentiating their unfolding. Biochem J.

[79] Kudryashova E, Seveau SM, Kudryashov DS (2017). Targeting and inactivation of bacterial toxins by human defensins. Biol Chem.

[80] Fischer S, Ückert AK, Landenberger M, Papatheodorou P, Hoffmann-Richter C, Mittler AK, Ziener U, Hägele M, Schwan C, Müller M, Kleger A, Benz R, Popoff MR, Aktories K, Barth H (2020). Human peptide α-defensin-1 interferes with *Clostridioides difficile* toxins TcdA, TcdB, and CDT. FASEB J.

[81] Korbmacher M, Fischer S, Landenberger M, Papatheodorou P, Aktories K, Barth H (2020). Human α-Defensin-5 efficiently neutralizes *Clostridioides difficile* toxins TcdA, TcdB, and CDT. Front Pharmacol.

[82] Barthold L, Heber S, Schmidt CQ, Gradl M, Weidinger G, Barth H, Fischer S (2022). Human α-defensin-6 neutralizes *Clostridioides difficile* toxins TcdA and TcdB by direct binding. Int J Mol Sci.

[83] King MC, Wilson AC (1975). Evolution at two levels in humans and chimpanzees. Science.

[84] Carroll SB (2005). Evolution at two levels: on genes and form. PLoS Biol.

[85] Bayer TS, Booth LN, Knudsen SM, Ellington AD (2005). Arginine-rich motifs present multiple interfaces for specific binding by RNA. RNA.

[86] Futaki S, Nakase I, Tadokoro A, Takeuchi T, Jones AT (2007). Arginine-rich peptides and their internalization mechanisms. Biochem Soc Trans.

[87] Helsen J, Voordeckers K, Vanderwaeren L, Santermans T, Tsontaki M, Verstrepen KJ, Jelier R (2020). Gene loss predictably drives evolutionary adaptation. Mol Biol Evol.

[88] Seto Y, Tamura K (2013). Extensive differences in antifungal immune response in two *Drosophila* species revealed by comparative transcriptome analysis. Int J Genomics.

[89] Hanson MA, Hamilton PT, Perlman SJ (2016). Immune genes and divergent antimicrobial peptides in flies of the subgenus *Drosophila*. BMC Evol Biol.

