# Supplementary material for: The evolutionary novelty of insect defensins: from bacterial killing to toxin neutralization
Source: Cell Mol Life Sci. 2024 May 23;81(1):230. doi: 10.1007/s00018-024-05273-5 (PMC11116330; doi:10.1007/s00018-024-05273-5)
Supplement: Supplementary file 1 — Supplementary Material 1 [file 18_2024_5273_MOESM1_ESM.pdf]

CMLS

Supplementary Information for

The evolutionary novelty of insect defensins: from bacterial killing to toxin neutralization

Bin Gao and Shunyi Zhu

E-mail: Zhusy@ioz.ac.cn

This PDF file includes:

Figures S1–S12.

Tables S1–S3.

A

κB, GATA, TATA box

tcacctatt**GAAAGTCCCC**taacttatctgaatg**AGATAG**tttacgattcggg**TATAAA**aactctt

Inr, DPE

gttacattgtcga**TCA**acacattacaaagtgcctagcccagcagctgcataga**AGTCC**taaccaac

taaaggaaaccctcatccaaagtcaacaaca

B

κB, GATA

tccccccag**GGGAACTCCC**ttagcttgttgctgctgagactctgatgttaagcct**TGATAG**agcca

TATA box, Inr

agcctgtggcg**TATAAA**agccagctgtagcttccgagcaca**TCATTC**agtgacagtgacagagc

DPE

AGTTCaagcaag

Fig. S1. Core and proximal promoter sequences of *DvirARP* (A) and *DvirDEF* (B). Transcription factor (TF) binding sites are capitalized in *red* and boxed; transcription initiation sites in Inr (initiator) are underlined twice.

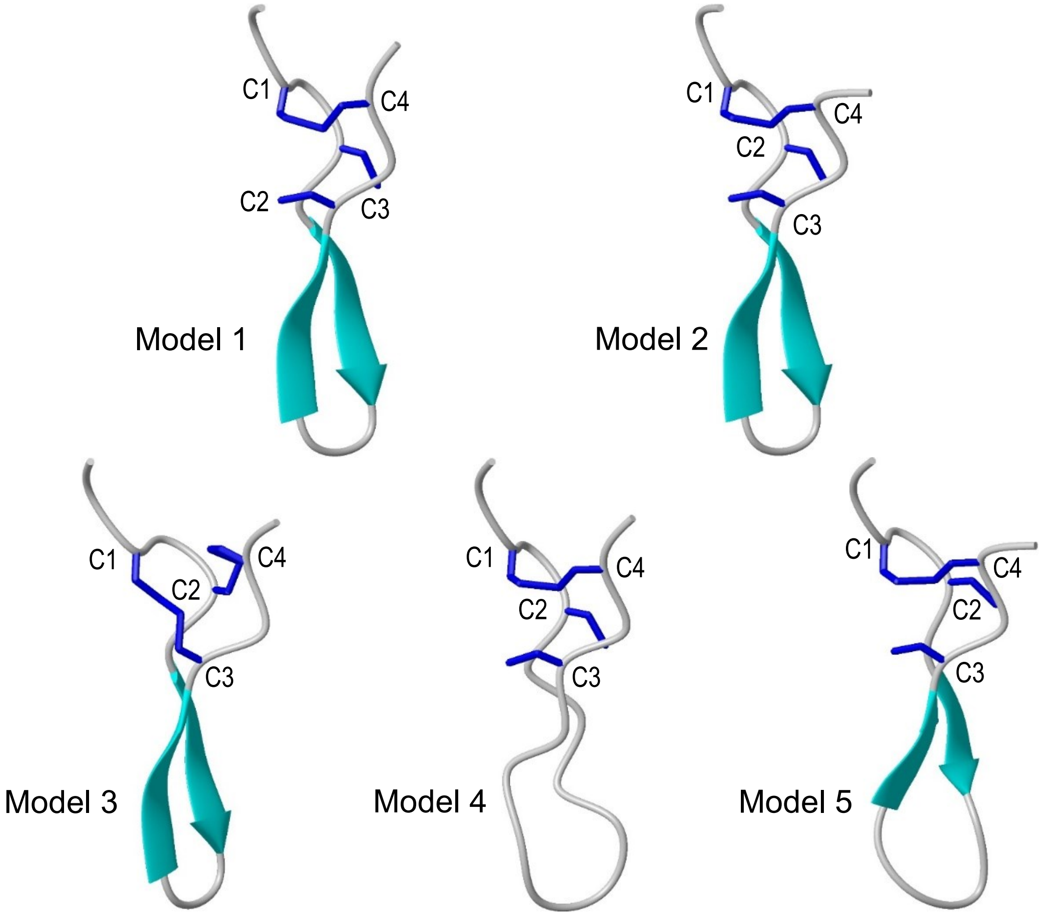

Fig. S2. Computational models of *Dvir*ARP. Five top models (models 1 -5) built by trRosetta (http://yanglab.qd.sdu.edu.cn/trRosetta/) based on *de novo* folding, guided by deep learning restraints, are displayed as ribbon diagram, with predicted disulfide bridges and free cysteine residues shown as *blue* sticks. The confidence of these models are low, with an estimated TM-score ≤ 0.295.

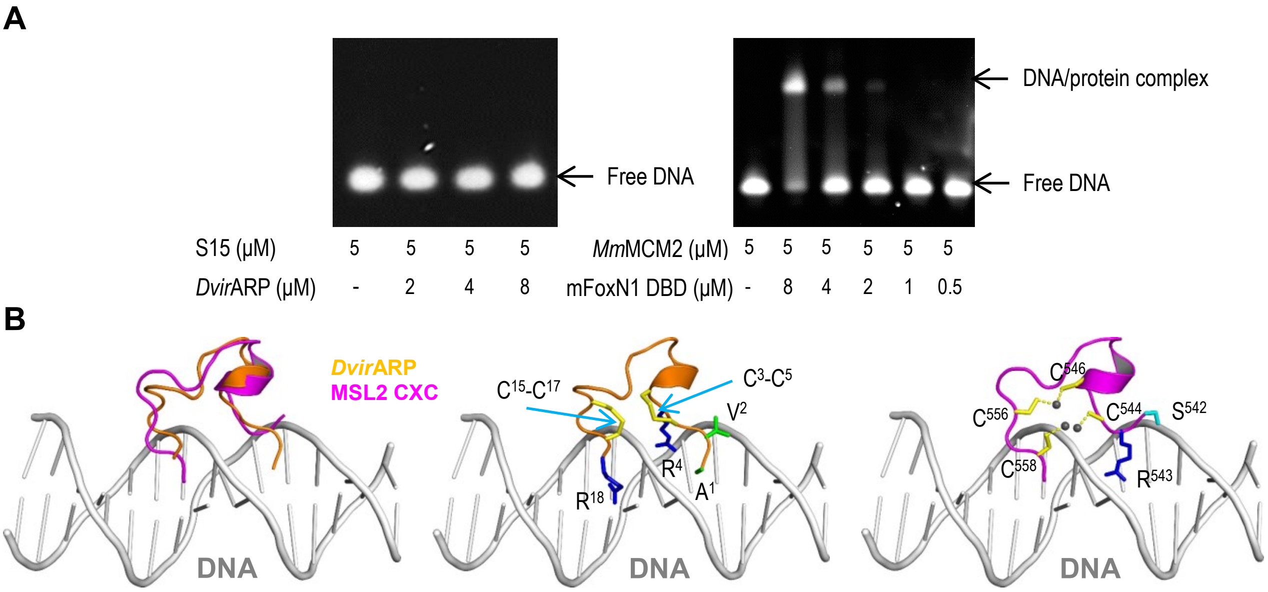

Fig. S3. *Dvir*ARP is not a DNA binder. (**A**) Electrophoretic mobility shift assays. S15 probe and *Dvir*ARP reaction (*Left*). *Mm*MCM2-2 probe and mFOXN1 DNA binding domain (DBD) reaction (*right*). "-" denotes no *Dvir*ARP or mFoXN1 DBD added. Note: mFOXN1 DBD was purified from the thrombin-digested product of a fusion protein carrying the GST tag by RP-HPLC using a semi-preparative C_{18} column. (**B**) Structural superimposition of *Dvir*ARP with the MSL2 CXC domain in complex with S15 DNA (pdb entry 4RKH) (*Left*). In a similar position to the MSL2 CXC domain on the DNA (middle), two arginine residues (R^4 and R^{18}) of *Dvir*ARP, indicated by *blue* sticks, respectively insert into the major and minor grooves of the DNA. Two disulfide bridges are denoted by *cyan* arrows. In this model, two hydrophobic residues (A^1 and V^2), shown by *green* sticks, make a steric hindrance hampering DNA binding. On the contrary, in the experimental complex (*right*), two equivalent residues (S^{542} and R^{543}), shown by sticks, are involved in direct interactions with the DNA, in which R^{543} inserts vertically deep into the minor groove. Zn-S coordination bonds are represented by *yellow* dotted lines and three Zn ions are denoted by *grey* dots.

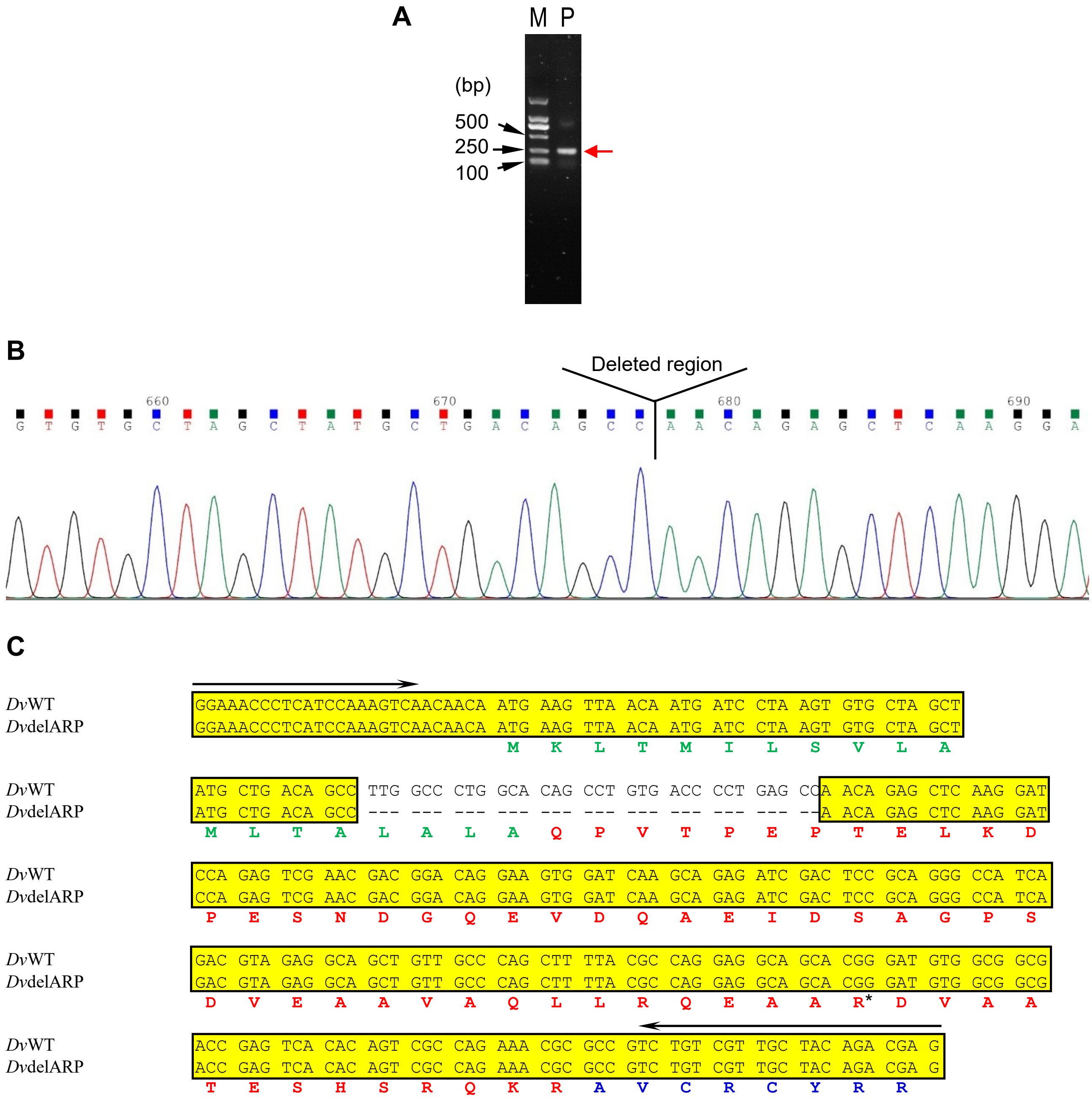

Fig. S4. Verification of *DvirARP* knockout (KO) mutant by PCR and DNA sequencing. (**A**) PCR amplification of the genomic DNA extracted from the *Dv*delARP mutant fly. The primer positions are shown in panel C. M, DNA marker; P, PCR product. (**B**) DNA sequencing map. The position of the deletion region leading to a frame-shift mutation is shown. (**C**) Sequence comparison between Dvir*ARP* and its mutant, in which the deleted bases are denoted by "-'. Identical bases are boxed and shaded *yellow*. The polymorphic site different from the genomic sequence retrieved from the GenBank database is marked by an asterisk. Arrows represent positions of the two primers (*Dv*delARP-F and *Dv*delARP-R).

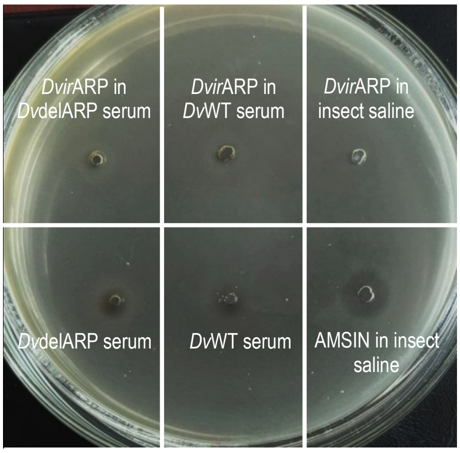

Fig. S5. Inhibition zone assay of *Dvir*ARP against *S. aureus* CGMCC 1.89 in different conditions. *Dvir*ARP was dissolved in insect saline (200 mM NaCl, 3.1 mM KCl, 5.4 mM $CaCl_2$, 5 mM $MgCl_2$, 2 mM $NaHCO_3$, and 0.1 mM NaH_2PO4, pH 7.2) or 50% haemolymph serum collected from *Dv*WT or *Dv*delARP female adults. The peptide dose used was 1 nmol/well. AMSIN [60] dissolved in insect saline (0.5 nmol/well) and the full serums alone were used as controls.

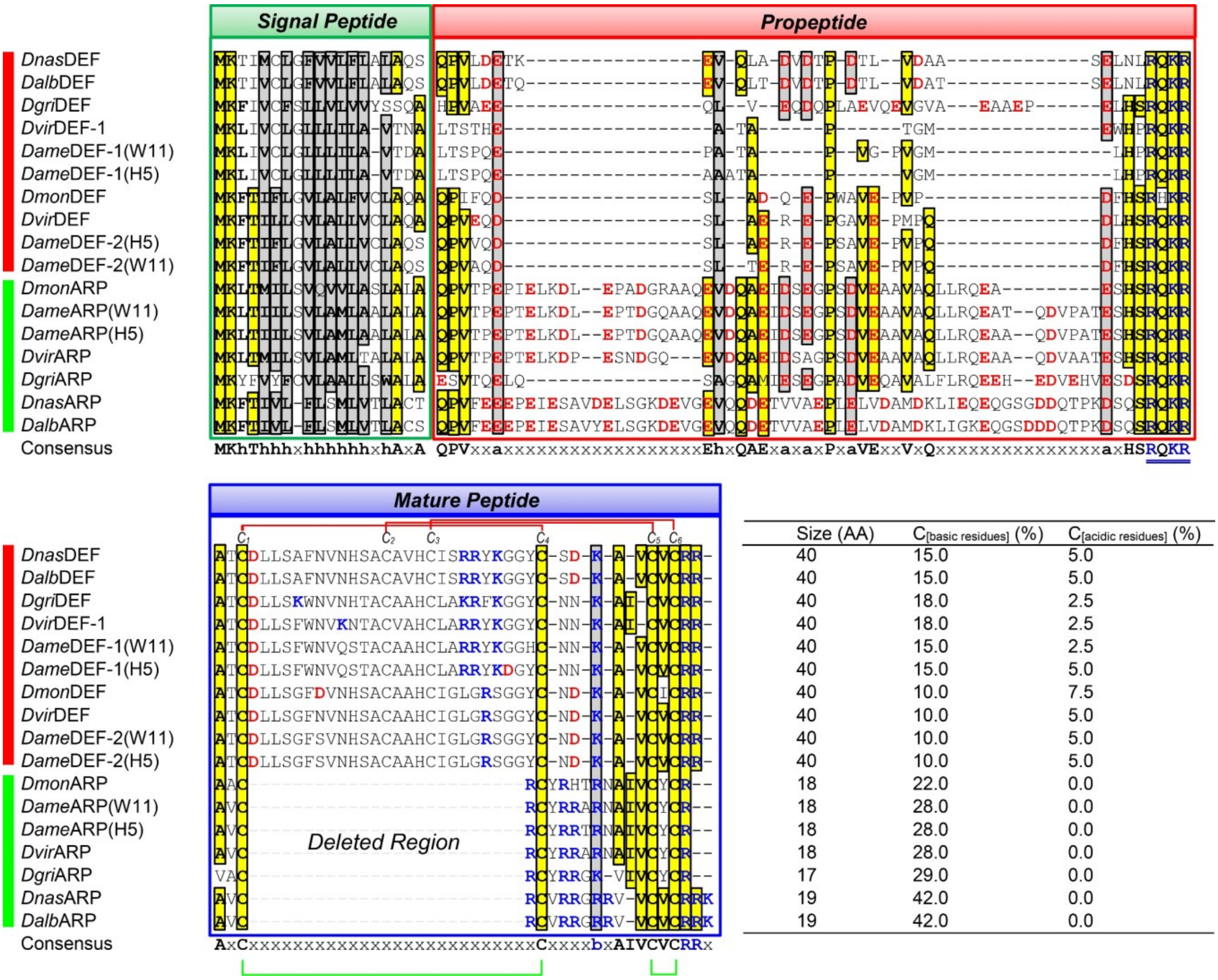

Size (AA)	$C_{[basic\ residues]}$ (%)	$C_{[acidic\ residues]}$ (%)
40	15.0	5.0
40	15.0	5.0
40	18.0	2.5
40	18.0	2.5
40	15.0	2.5
40	15.0	5.0
40	10.0	7.5
40	10.0	5.0
40	10.0	5.0
40	10.0	5.0
18	22.0	0.0
18	28.0	0.0
18	28.0	0.0
18	28.0	0.0
17	29.0	0.0
19	42.0	0.0
19	42.0	0.0

Fig. S6. Precursor sequence comparison between insect defensins and ARPs from the *Drosophila* subgenus. Insect defensins and ARPs are marked by *red* and *green* bars,

respectively. Gaps are introduced to maximize the alignment. Signal peptides, propeptides and mature peptides are boxed in *green*, *red* and *blue*, respectively, in which identical and conserved residues are boxed and shadowed in *yellow* and *grey*, respectively. According to this conservation, a consensus is provided here, in which "a, b, h and x" represent acidic, basic, hydrophobic and any amino acids, respectively. In the propeptides, negatively charged residues are colored in *red*, with a Kex2-like processing endoprotease cleavage motif (RQKR) is underlined twice in *blue*. In the mature peptides, positively and negatively charged residues are colored in *blue* and *red*, respectively. The evolutionarily deleted region in ARPs is indicated here. Molecular sizes and the content percentages of basic and acidic residues in each mature peptide are shown at the right-hand corner of this figure. Abbreviations for species names: *Dnas*, *Drosophila nasuta*; *Dalb*, *Drosophila albomicans*; *Dgri*, *grimshawi*; *Dvir*, *Drosophila virilis*; *Dame*, *Drosophila americana*; and *Dmon*, *Drosophila montana*.

	Signal Peptide	Propeptide	Mature Peptide
*Dnav*DEF-1	MK--FIAYLGILLAVVVYLVQA	QPLEQDSLD---GREP--------GAVEPTQQESHSRLKR	ATCDLLSGFSVNHSACAVHCIGLGKSGGYCNDKAVCVCRR
*Dmoj*DEF-2	MK--FIVYLGVLLAVVVYFAQA	QLLPQDSLD---EREP--------GAVEPTQQELHSRLKR	ATCDLLSGFSVNHSACAVHCIGLGKSGGYCNDKAVCVCRR
*Dele*DEF	MK--FIVFLAVALAFVAVVIQA	QPVPEDES---APVDH----PLVSQDAQEGALVEHSRQKR	ATCDLLSKWNWNHTACAAHCVTKGFKGGYCSDKAVCVCRR
*Drho*DEF	MK--FIVFLAIALALVAVVVQA	QPVPEEDH---IPAEH----PLVEQEA-------HSRQKR	ATCDLLSKWNWNHTACAAHCLTKGFKGGYCSDKAVCVCRN
*Dsec*DEF	MK--FFVLVAIAFALLACMAQA	QPVSDVDP---IPEDH----ALVHEDAH-QEVVQHSRQKR	ATCDLLSKWNWNHTACAGHCIAKGFKGGYCNDKAVCVCRN
*Dsim*DEF	MK--FFVLVAIAFALLACMAQA	QPVSDVDP---IPEDH----ALVHEDAH-QEVVQHSRQKR	ATCDLLSKWNWNHTACAGHCIAKGFKGGYCNDKAVCVCRN
*Dmel*DEF	MK--FFVLVAIAFALLACVAQA	QPVSDVDP---IPEDH----VLVHEDAH-QEVLQHSRQKR	ATCDLLSKWNWNHTACAGHCIAKGFKGGYCNDKAVCVCRN
*Dyak*DEF	MK--FFVFVAIVFALLACMVQA	QPVSDVDS---ISEDH----PLVHEDAH-QEVVQHSRQKR	ATCDLLSKWNWNHTACAGHCIAKGFKGGYCNDKAVCVCRN
*Dere*DEF	MK--FFAFVAIAIALLACVVQA	QPVSDVDP---TPEDH----PLVHEAAH-QEVVQHSRQKR	ATCDLLSKWNWNHTACAGHCIAKGFKGGYCNDKAVCVCRN
*Deug*DEF	MK--FFAFLAIALALVACMVQA	QPVSDVET---YPAEN----PLLIKDPQ-QEVVEHSRQKR	ATCDLLSKWNWNHTACAGHCIAKGFKGGYCNDKAVCVCRN
*Dbia*DEF	MK--FLVFLAIALALVACLAQA	QPVPEEDQ---PAATN----PLVHEDVH-QEVVEHSRQKR	ATCDLLSKWNWNHTACAAHCIAKKYKGGYCNDKAVCVCRN
*Dtak*DEF	MK--FVVFLAICLALVACLVQA	QPVPEEDQ---NPADH----PLVNQDVH-EEV-QHSRQKR	ATCDLLSKWNWNHTACAAHCIAKRFKGGYCSDKAVCVCRN
*Dana*DEF	MK--VIVFCALALVLVS-VVQA	H---VKS----LDVHT----LEKDLPGN-ADVQEHSRQKR	ATCDLLSKWNWNHTACAAHCIARKFKGGYCNDKAVCVCRH
*Dbip*DEF	MK--VMVFLALALVLVG-VVQA	QPVADKSPKEVLEVHT----LEEDLPEN-ADVQEHSRQKR	ATCDLLSKWNWNHTACAAHCLARKYKGGYCNDKAVCVCRH
*Dfic*DEF	MK--IFALLAFAMVLMACMVQA	QPVAVVEN---YPAGS----LLDIQDAH-QQVVQHSRQKR	ATCDLLSKWNWNHTACAAHCVARRFKGGYCNDKAVCVCRK
*Dlac*DEF-3	MNSLWYSLLAIALALVACLVQA	QPVPEEALEESLLAE----------QQPDLALVEHSRQKR	ATCDLLSKWNWNHTACAAHCLARGNKGGYCNDKAVCVCRR
*Dasa*DEF-3	MNSLWYSLLAIALALVACLVQA	QPVPEEDLEEILLAE----------QQPDLALVEHSRQKR	ATCDLLSKWNWNHTACAAHCLARGNKGGYCNDKAVCVCRR
*Daur*DEF-1	MK--FIVFLAIALALVACLVQA	QPVPEEALEESLLEE----------QQPDLALVEHSRQKR	ATCDLLSKWNWNHTACAAHCLARGNKGGYCNDKAVCVCRR
*Dtri*DEF-1	MK--FSVFLAIALALVACLVQA	QPVPEEALEESLLEE----------QQPDLALVEHSRQKR	ATCDLLSKWNWNHTACAAHCLARGNKGGYCNDKAVCVCRR
*Druf*DEF-1	MK--FIVFLAIALVLVACLVQA	QPVPEEALEDSLLAE----------QQPDLALVEHSRQKR	ATCDLLSKWNWNHTACAAHCVARGNKGGYCNDKAVCVCRR
*Dasa*DEF-1	MK--FIVFLAIALALEACLVQA	QPVPEEALEQSLLEE----------QQPDLALVEHSRQKR	ATCDLLSKWNWNHTACAAHCVARGNRGGYCNDKAVCVCRR
*Dtan*DEF-1	MK--FIVFLAIALALVICLVQA	QPVPEEALVNSLLAE----------QQPDLALVEHSRQKR	ATCDLLSKWNWNHTACAAHCLARGNKGGYCNDKAVCVCRR
*Dkik*DEF-2	MK--IILFLALAFAFVACLVQA	QPVKEEALPEHLLAEAVEQPDQATEEQADQGVAEHSRQKR	ATCDLLSKWNWNHTACAAHCLARGNKGGYCNDKAVCVCRR
*Dlac*DEF-1	....	....	....................AHCLARGNKGGYCNDKAVCVCRR
*Dper*DEF	MK--FIVFLALSLA-VMCLVQA	QPLAEEPIEEDVAKP---LVQLMEQPVQDVDVPEHSRQKR	ATCDLLSKWNVKHTACAGHCLAKGFKGGYCNNKAVCICRR
*Dpse*DEF	MK--FIVFLALSLA-VMCLVQA	QPLAEEPIEEDVAKP---LVQLMEQPVQDVDVPEHSRQKR	ATCDLLSKWNVKHTACAGHCLAKGFKGGYCNNKAVCICRR
*Daur*DEF-2	MK--LIVFLALALAFVACVAQA	QPVTELALD-----------------EVALEEVPHSRQKR	VTCDLLSKWNWNDTACAAHCLLKGKRGGHCSSNGVCVCR-
*Dtri*DEF-2	MK--LIIFLALALAFVACVAQA	QPVTELALE-----------------EVALEEVPHSRQKR	VTCDLLSKWNWNDTACAAHCLLTGKRGGHCSSNGVCVCR-
*Dtan*DEF-2	MK--LTIFLAFALGLVACVAKA	QPVTELAL----------------------EDVPHSRQKR	FTCDLLSGLNWNNALCAAHCLLTGKRGGYCSNKGVCICR-
*Dasa*DEF-2	MK--LIVFLALALASLACVAKA	QPVTALAL----------------------MDEPHSRQKR	FTCDLLSKWNWNHTACAAHCLTLGKSGGYCSDKAVCICR-
*Dasa*DEF-4	MK--LIVFLALALASLACVAKA	QPVTMLAL----------------------MDEPHSRQKR	FTCDLLSKWNWNHTACAAHCLTLGKSGGYCSDKAVCICR-
*Druf*DEF-2	MK--LIVFLALALASLACVAKA	QPVTMLAL----------------------VDEPHSRQKR	FTCDLLSKWNWNHTACAAHCLTLGKSGGYCSDKAVCICR-
*Dlac*DEF-2	MK--LIIFLALALASLACVAKA	QPVTALAL----------------------VDEPHSRQKR	FTCDLLSKWNWNHTACAAHCLTLGKSGGYCSDKAVCICR-
*Dkik*DEF-1	MK--LILFLAFTLALVACLVQA	QPVTIRNLD-------------------QALVPHSRQKR	ATCDLLSGLNYNHSLCAAHCIALLKKGGYCTDKGVCVCRS
*Dmoj*DEF-1	MK--SIVCL--TLLVVVTLAIA	QPVVKETVE---VKEDFVVSPAALEEVQPAAPVAHQRYKR	ATCDLLSFLNVNNSACAVHCLAKRYKGGYCNSKAVCVCRN
*Dari*DEF	MK--SIVCL--TLLVVVTLAIA	QPLVKETVD---IKED-VVSPAALEEVQPAAPVTHQRYKR	ATCDLLSFLNVNNSACAVHCLAKRFKGGYCNSKAVCVCRH
*Dnav*DEF-2	MK--SIVFL--TLLVVVTMAIA	QPVVKETFE---VKEK-AVSLADLEEEQPAAPVDHQRYKR	ATCDLLSFLNVSHAACTAHCLLKRFKGGYCNSKAVCVCRN
Consensus	MKxxhhhhhAhhhhhhhChhQA	QPVxxxxxxxxxxxxxxxxxxxxxxxxxxxxxxxxxHSRQKR	ATCDLLSKWNWNHTACAhHChhbGxbGGYCpDKAVChCRx

Fig. S7. Alignment of precursor sequences of *Drosophila* defensins. Signal peptides, propeptides and mature peptides are boxed in different colors. All sequences are derived from the *Sophophora* subgenus except the last three from the *Drosophila* subgenus. Identical and conserved residues are shadowed in *yellow* and *grey*, respectively. In the end of the propeptide region, a Kex2-like processing endoprotease cleavage motif (RXKR, X denoting any amino acids) is underlined twice in *blue*. Gaps are introduced to maximize the alignment and dots represent amino acids not available. In the consensus, 'a, b, h, p, and x' represents acidic, basic, hydrophobic, polar and any amino acids, respectively.

	Signal Peptide	Propeptide	Mature Peptide
*Dlac*DLP-2	-MK-ITI---FLALTLA-ALYMVKA	DPLNREEAQGHPLEQDQDE--IYLEP-IQQDRLKR	-ANCDAGRCNQICAT-RGKMGLCRKG-NCKCY-
*Dasa*DLP-2	-MK-IPI---FLALTLA-ALYMVKA	DPLNREEAQGHPLEQDQDE--IFLEP-IQQDRLKR	-ANCDAGRCNQICAT-RGKMGLCRKG-NCKCY-
*Druf*DLP-1	-MK-ITI---FLALTLA-ALYMVKA	DPLNREEAQGHPLEQDQDD--IYLEP-IQQDRLKR	-ANCDVGQCNRICAT-RGKMGLCRKG-NCKCY-
*Dtan*DLP-3	-MK-FTV---FLALTLA-ALYVAHG	DSLNKKEAQGHPLEQEQDEGNIFLEP-IQQDRLKR	-ANCDVGRCNQICAT-RGKMGLCRKG-NCKCY-
*Daur*DLP-2	-MK-ITI---FLALTLA-ALYVAQA	Q---GQEAQGHLLEQEQDEGNIGLEKPSQQHRLKR	-ANCDVGRCNRICAT-RGKMALCRKG-ICKCY-
*Dtri*DLP-2	-MK-ITI---FLALTLA-ALYVAQA	Q---GQEAQGHFLEQEQDEGNIGLEKPSQQHRLKR	-ANCDVGRCNRICAT-RGKMGLCRKG-ICKCY-
*Dtan*DLP-1	-MK-ITI---FLALTLA-ALYVAHA	DPLHKEEAQGHPLKQEQDNGNFFLEP-TQLGRLKR	-ATCQTLQCAATCAR-QKRLGVCKKM-ECQCYQ
*Dtan*DLP-4	-MK-IRI---FLALNLA-AIYVAQG	DPLNKE--KGHPVEQEQDKGNIFLES-IQKDRLKR	-AACTNVGCIRFCGR-E-RLGVCRNG-GCVCG-
*Dasa*DLP-3	-MK-ITI---LIALTLA-GIHVGQA	DPLNKEEAQGHPLEQEQDKGNIFLEP-IEKDRLKR	-AACTNVGCIRFCGR-E-RLGVCRNG-GCVCG-
*Druf*DLP-2	-MK-IAI---LLALTLG-AVYVAQA	HPQNKE--EGYLVEQEQNEGNIFLRP-IEQDRLKR	-AACTNVGCIRFCDG-Q-TLGACRNG-GCECG-
*Druf*DLP	-MHTLCTGYALLALTLA-AVYVAQA	DPLNKE--EGYPVEQEQDEGNIFLKP-IEQDRLKR	-AACTNVRCIPLCDG-Q-TLGACRNG-GCVCG-
*Dkik*DLP	-MK-ITF---FLALVLA-AVYVSQA	EPLHKEDTQGFNLEEVQDTGSAFREL-SQERRLKR	-ATCAIDQCSAFCGK-KGRSALCRKG-HCKCY-
*Dele*DLP	-MK-ITI---ILALALV-AVCAVLA	EPATKGPNDVGSVNHYVDEQIPTEEL-KEHNRQKR	-ATCRINDCMSFCKK-RGTSGVCVKG-NCHCY-
*Drho*DLP	-MK-ITI---ILALALL-AVCAVLA	DPDTKVPNEVDPVNLDVDLGSSTQGL-EKHNLQKR	-ANCQIERCLAICRT-RSLLGVCVKG-KCRCQ-
*Dyak*DLP	-MK-ITI---ILALTLA-AICVMQV	QSIPEDPANG-LLQRELQ-RNNIQDA-EEHVRQKR	-QTC-IERCNPFCLK-KGFKGVCVKD-LCRCV-
*Dere*DLP	-MK-ITT---ILALILA-TICV--V	QSIPEDPDKG-LLQKELQ-GNLIQDS-EEHVRPKR	-QSC-AERCNSLCLK-KGLKGVCVKD-LCRCA-
*Dsec*DLP	-MK-ITI---ILALTLV-AVCVMQV	RSIPEDSENR-QLQKELQ-GSLIQDA-EEHVRPRR	-QSC-IERCNSLCSK-KGVRGVCVKG-LCRCT-
*Dsim*DLP	-MK-ITI---ILSLTLV-AVCVMRV	RSIPEDSENR-HLQKELQ-GSLIQDA-EEHVRPRR	-QSC-IERCNSLCSK-KGFRGVCVKG-LCRCT-
*Dmel*DLP	-MK-ITI---ILALTLV-AVCLMQV	RSIPKDSENR-LLQKELQ-RSLIQDA-KEHVRPRR	-QSC-IERCNSLCSK-KGFKGVCVKG-LCRCT-
*Deug*DLP	-MK-ITV---ILALTLA-AICVMQA	ETIPEDPP----LQQELQ-ENLFQDM-REQVRQKR	-ANCQIEKCTANCST-RGLKGVCIKGRKCRCY-
*Dbia*DLP	-MK-IAI---ILAVALA-AIGALQA	QSIPQNP-----LQQDLQ-RNLIQDG-ETHARQKR	-ADCNITNCSGICSR-RGLKGYCSKS-HCRCA-
*Dfic*DLP	-MK-IKF---ILALILA-AICVMQA	QDIPQEAKDQ-QLWKGLS-GNPIQEV-NVRSR-RR	TDICETWDCLLFCFDFSRGIGICVNG-FCHCT-
DtakDLP	-MK-ITI---ILTLIFA-AICVLQT	QTISEDPDNQ-PLQQDLQ-GNPIQDP----NRQIR	-ANCNTLSCSATCAK-QRLKGICLKG-TCRCFH
*Daur*DLP-1	-MK-LIV---FLALALALVACLAQA	QPVAEVALEEVALEEV------------PHSRQKR	-ARCDLSACTDFCILFKNRGGCCALG-YCTCR-
*Dtri*DLP-1	-MK-LIV---FLAIALAMMACLIKA	QSVTEVALDAV-----------------PHSRQKR	-ARCEHSACNDLCIAVKRRGGYCVLG-FCTCR-
*Dlac*DLP-1	MMK-FIV---FLALVLAMMACLAKA	QSVTEVALEAV-----------------PHSRQKR	-ASCDPGGCAAHCLLSGKRMGFCLLG-YCNCR-
*Dasa*DLP-1	MMK-FIV---FLALVLAMMVCLAKA	QSATEVALEAV-----------------PHSRQKR	-ASCDPGGCAAHCLLSGKRMGFCLLG-YCNCR-
*Dtan*DLP-2	MMK-FIV---FLALVLAMMACLAKA	QSVTEVALAPVYVVKAQPLNNEEAQGHALELEQHI	-AYCNVGQCNRNCAIEGK-SGHCSKKAKCKCY-
Consensus	xMKxhxhxxxhLAhxLhxhhxhhxh	xxxxxxxxxxxxxxxxxxxxxxxxxxxxxxxRxKR	xxxCxxxxCxxxCxxxxxxxGxCxxxxxCxCxx

Fig. S8. Alignment of precursor sequences of *Sophophora* defensin-like peptides (DLPs). The explanatory notes are the same as those in Fig. S7. The free cysteine in *Daur*DLP-1 is shown in *red*.

A

		PDB Entry
*Dtri*DLP-1	ARCEHSACNDLCIAVKRRGGYCVLGFCTCR	-
Tc32	TTCQAAMCEAGCKGLGKSMESCQGDTCKCK	2JP6
DEF-AAA	VTPNDSLCAAHCLVKGYRGGYCKNKICHCR	2E3E
Defensin1	MACQFWSCNSSCISRGYRQGKCWYKYCQCY	6BB6
Coprisin	IAVNHSACALHCIALRKKGGSCQNGVCVCR	2LN4
Racemic Plectasin	WDEDDMQCHNHCKSIGYKGGYCAGGVCKCY	3E7R

B

		PDB Entry
*Dtri*DLP	ATCDLSACTDFCILFKRRGGCCVLGYCTCR	-
DEF-AAA	ATCDLSLCAAHCLVKGYRGGYCKNKICHCR	2E3E
Dg3b	ITCDLRLCVVHCLAKGFRGGWCDRKVCNCR	6PX8
Coprisin	VTCDVSACALHCIALRKKGGSCQNGVCVCR	2LN4
DEF-ABB	ATCDLSLCAAHCLVKGYRGGYCKNKICHCR	2NY9
Lucifensin	ATCDLSACAAHCLLRGNRGGYCNRAICVCR	2LLD

Fig. S9. Homologous templates for building models of *Dtri*DLP-1 (A) and *Dtri*DLP (B). These templates were recognized by trRosetta (https://yanglab.nankai.edu.cn/trRosetta/). In the alignment, identical and conserved residues to the queries are shadowed in *yellow* and *grey*, respectively.

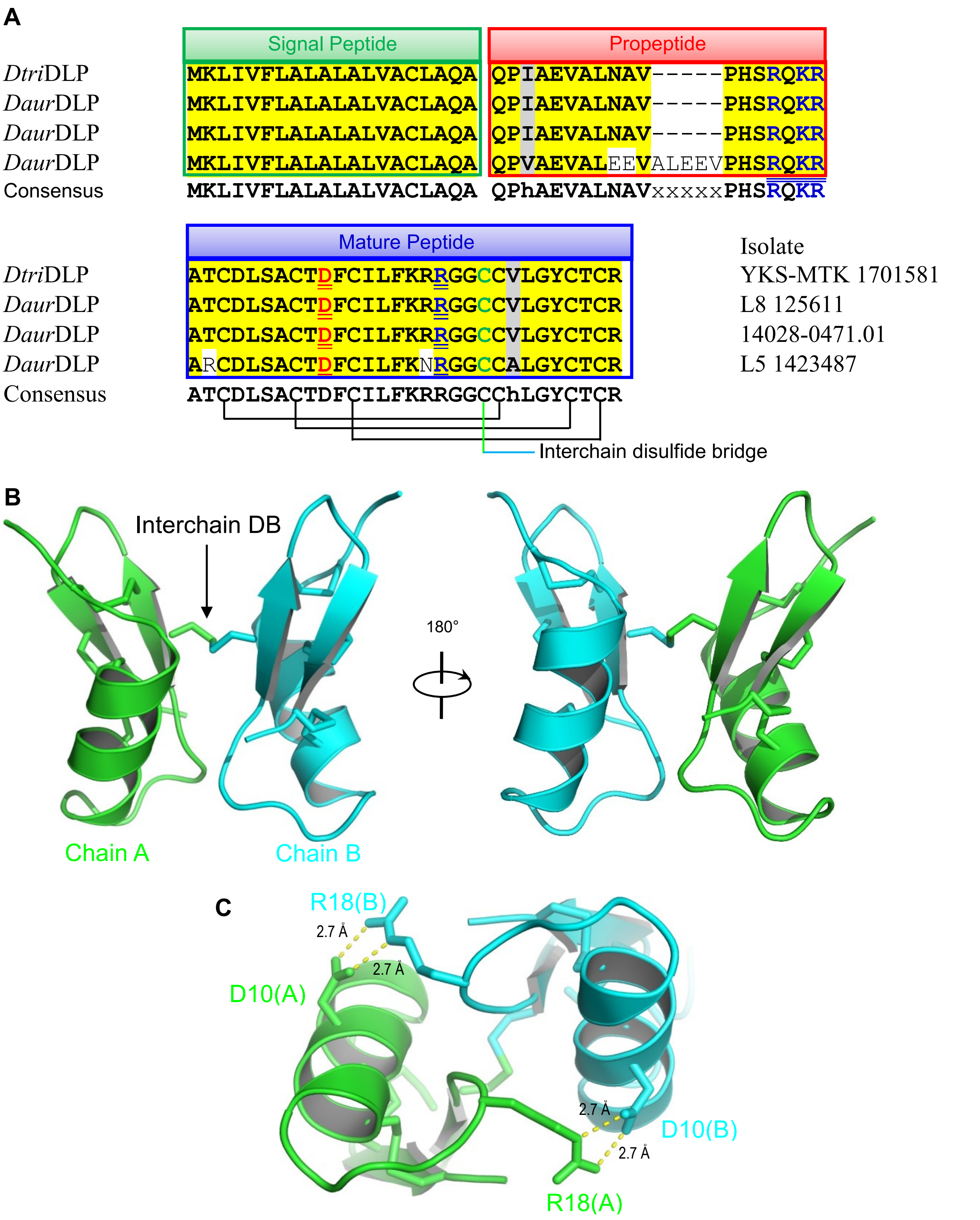

Fig. S10. The DLPs with an odd number of cysteines from *Drosophila triauraria* (isolate YKS-MTK 1701581) and *D. auraria* (multiple isolates). (**A**) Precursor sequence alignment. The explanatory notes are the same as those in Fig. S7. The cysteines proposed to form an interchain disulfide bridge are colored *green*. The two residues involved in the salt bridge formation in the dimer model are underlined twice and colored *red* for Asp and blue for Arg. (**B** and **C**) The computational model of *Dtri*DLP showing the position of the interchain disulfide bridge and the two salt bridges formed between Asp-10 and Arg-18 derived from each chain, in which hydrogen bonds are indicated by *yellow* dotted lines with their distances shown.

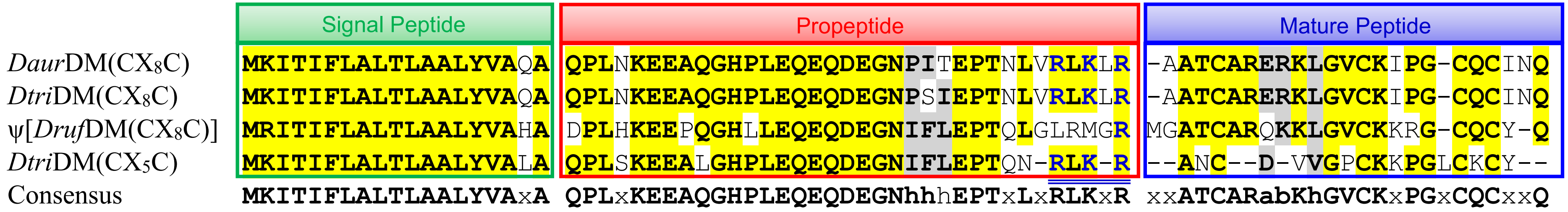

Fig. S11. Alignment of precursor sequences of *Sophophora* CX_5C and CX_8C deletion mutants. The explanatory notes are the same as those in Fig. S7.

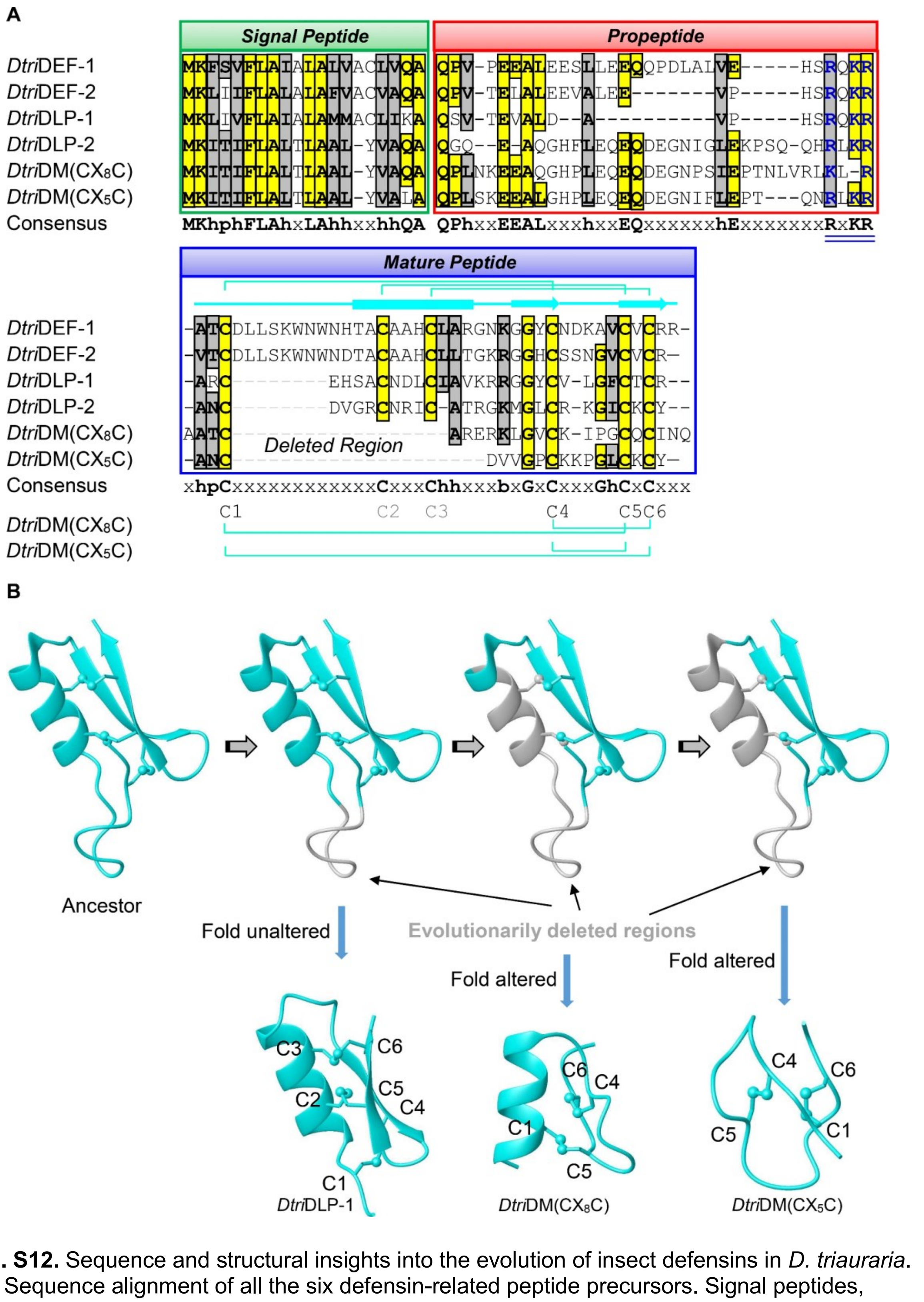

Fig. S12. Sequence and structural insights into the evolution of insect defensins in *D. triauraria*. (**A**) Sequence alignment of all the six defensin-related peptide precursors. Signal peptides, propeptides and mature peptides are boxed in *green*, *red* and *blue*, respectively, in which identical and conserved residues are shadowed in *yellow* and *grey*, respectively. In the end of the propeptide region, a Kex2-like processing endoprotease cleavage motif (RXKR, "X" denoting any amino acids) is colored *blue* and underlined twice. Secondary structural elements and cysteine pairings for disulfide bridge formation are extracted from the structures described here. (**B**) Structure models showing a gradual truncation trend in the evolution of an ancestral defensin, leading to fold type alteration accompanying disulfide bridge reorganization.

Table S1. Primers used in this study.

Name	Sequence (5'-3')	Usage
dT3AP	CTGATCTAGAGGTACCGGATCCTTTTTTTTTTTTTTTTT	Reverse transcription
3AP	CTGATCTAGAGGTACCGGATCC	RT-PCR
InsectRP49	AAGGGHCARTAYYTKATGCC	Semi-Quantitative RT-PCR
*Dvir*DEF-F	GATGAAATTTACCATACTCCT	cDNA and genomic cloning; Semi-Quantitative RT-PCR
*Dvir*ARP-F	AACAATGATCCTAAGTGTGCT	cDNA cloning; Semi-Quantitative RT-PCR
*Dvir*ARP-R	TTGGTTTACTATAAAGACGCCCT	cDNA and genomic cloning; Semi-Quantitative RT-PCR
*Dv*delARP-F	GGAAACCCTCATCCAAAGTCA	Genomic cloning
*Dv*delARP-R	CTCGTCTGTAGCAACGACAGA	Genomic cloning

Note: H=A/C/T; R=A/G; Y=T/C; K=G/T.

Table S2. Sources and culture conditions of the microorganisms used in this study.

Strains	Sources	Culture conditions
**Gram-positive bacteria**		
Bacillus megaterium CGMCC 1.0459	Center for Microbial Resources, Institute of Microbiology, Beijing, China	LB, 37°C
Bacillus subtilis CGMCC 1.2428	Center for Microbial Resources, Institute of Microbiology, Beijing, China	ditto
Curtobacterium luteum	Isolation by our own lab and characterization by Institute of Microbiology, Beijing, China	TGY, 30°C
Lysinibacterium fusiformis	Isolation and characterization by our own lab [sequencing 16S rDNA]	Broth Medium, 37°C
Micrococcus luteus CGMCC 1.0290	Center for Microbial Resources, Institute of Microbiology, Beijing, China	LB, 37°C
Methicillin-sensitive *Staphylococcus aureus* (MSSA) CGMCC 1.89	Center for Microbial Resources, Institute of Microbiology, Beijing, China	Broth Medium, 37°C
Penicillin-sensitive *Staphylococcus epidermis* (PSSE) P1111	302nd Military Hospital, Beijing, China	ditto
Methicillin resistant coagulase-negative *Staphylococci* (MRCNS) P1369	302nd Military Hospital, Beijing, China	ditto
Methicillin-resistant *Staphylococcus aureus* (MRSA) P1374	302nd Military Hospital, Beijing, China	ditto
Penicillin-resistant *Staphylococcus aureus* (PRSA) P1383	302nd Military Hospital, Beijing, China	ditto
Methicillin-resistant *Staphylococcus aureus* (MRSA) P1386	302nd Military Hospital, Beijing, China	ditto
Penicillin-resistant *Staphylococcus epidermis* (PRSE) P1389	302nd Military Hospital, Beijing, China	ditto
Staphylococcus aureus J685	Gifted by Dr. Jing Qi (Shandong Academy of Agricultural Sciences, Jinan, China)	ditto
Staphylococcus warneri ATCC 1.2824	Center for Microbial Resources, Institute of Microbiology, Beijing, China	ditto

Streptococcus mutans ATCC 1.2499	Center for Microbial Resources, Institute of Microbiology, Beijing, China	ditto
Streptococcus salivarius ATCC 1.2498	Center for Microbial Resources, Institute of Microbiology, Beijing, China	ditto
Streptococcus sanguinis ATCC 1.2497	Center for Microbial Resources, Institute of Microbiology, Beijing, China	ditto
**Gram-negative bacteria**		
Alcaligenes faecalis CGMCC 1.1837	Center for Microbial Resources, Institute of Microbiology, Beijing, China	Broth Medium, 37°C
Escherichia coli ATCC 25922 1.2385	Center for Microbial Resources, Institute of Microbiology, Beijing, China	LB, 37°C
Escherichia coli Am. J16c; Am. J23a; CIP. J14b; D. G2b; D. J45b	Gifted by Dr. Jing Qi (Shandong Academy of Agricultural Sciences, Jinan, China)	ditto
Escherichia coli DH5α	Preserved by our own lab	ditto
Pseudomonas aeruginosa O1; 14; FRD1	Gifted by Dr. Luyan Ma (Institute of Microbiology, Beijing, China)	Broth Medium, 37°C
Pseudomonas aeruginosa 374	Gifted by Prof. Yang Wang (China Agricultural University, Beijing, China)	ditto
Pseudomonas aeruginosa DH; QT1; 11082603; 11082616; 11092304; 11092618	Gifted by Dr. Jing Qi (Shandong Academy of Agricultural Sciences, Jinan, China)	ditto
Pseudomonas solanacearum	Gifted by Prof. Fengming Song (Zhejiang University, Hangzhou, China)	Broth Medium, 30°C
Salmonella enterica ATCC 14028	Center for Microbial Resources, Institute of Microbiology, Beijing, China	ditto
Serratia marcescens ATCC 14041	Center for Microbial Resources, Institute of Microbiology, Beijing, China	ditto
Stenotrophomonas maltophilia CGMCC 1.1788	Center for Microbial Resources, Institute of Microbiology, Beijing, China	ditto
**Fungi**		
Neurospora crassa CGMCC 3.1605	Center for Microbial Resources, Institute of Microbiology, Beijing, China	YPD, 28°C
Geotrichum candidum CCTCC AY 93038	China Center for Type Culture Collection, Wuhan University, Wuhan, China	ditto
Aspergillus nidulans A28	Gifted by Prof. Shaojie Li (Institute of Microbiology, Beijing, China)	ditto
Aspergillus fumigatus YJ-407	Gifted by Prof. Cheng Jin (Institute of Microbiology, Beijing, China)	ditto
Aspergillus flavus	Gifted by Prof. Yijian Yao (Institute of Microbiology, Beijing, China)	ditto
Aspergillus niger	Gifted by Prof. Yijian Yao (Institute of Microbiology, Beijing, China)	ditto
Beauveria bassiana 252	Gifted by Prof. Chunju An (China Agricultural University, Beijing, China)	ditto
Metarhizium robatsii 2575	Gifted by Prof. Weiguo Fang (Zhejiang University, Hangzhou, China)	ditto
Pichia pastoris X33	Gifted by Prof. Wenjun Liu (Institute of Microbiology, Beijing, China)	ditto

Table S3. Evaluation of the antimicrobial activity of *Dvir*ARP against various microorganisms.

Microorganism	C_L (μM)
**Gram-positive bacteria**	
Bacillus megaterium CGMCC 1.0459	17.68
Bacillus subtilis CGMCC 1.2428	N.A.
Curtobacterium luteum	N.A.
Lysinibacterium fusiformis	N.A.
Micrococcus luteus CGMCC 1.0290	10.77
Methicillin-sensitive *Staphylococcus aureus* (MSSA) CGMCC 1.89	N.A.
Penicillin-sensitive *Staphylococcus epidermis* (PSSE) P1111	N.A.
Methicillin resistant coagulase-negative *Staphylococci* (MRCNS) P1369	N.A.
Methicillin-resistant *Staphylococcus aureus* (MRSA) P1374	N.A.
Penicillin-resistant *Staphylococcus aureus* (PRSA) P1383	N.A.
Methicillin-resistant *Staphylococcus aureus* (MRSA) P1386	N.A.
Penicillin-resistant *Staphylococcus epidermis* (PRSE) P1389	N.A.
Staphylococcus aureus J685	N.A.
Staphylococcus warneri ATCC 1.2824	N.A.
Streptococcus mutans ATCC 1.2499	N.A.
Streptococcus salivarius ATCC 1.2498	N.A.
Streptococcus sanguinis ATCC 1.2497	N.A.
**Gram-negative bacteria**	
Alcaligenes faecalis CGMCC 1.1837	N.A.
Escherichia coli ATCC 25922 1.2385	N.A.
Escherichia coli Am. J16c; Am. J23a; CIP. J14b; D. G2b; D. J45b	N.A.
Escherichia coli DH5α	N.A.
Pseudomonas aeruginosa O1; 14; FRD1; 374; DH; QT1; 11082603; 11082616; 11092304; 11092618	N.A.
Pseudomonas solanacearum	N.A.
Salmonella enterica ATCC 14028	N.A.
Serratia marcescens ATCC 14041	N.A.
Stenotrophomonas maltophilia CGMCC 1.1788	N.A.
**Fungi**	
Neurospora crassa CGMCC 3.1605	N.A.
Geotrichum candidum CCTCC AY 93038	N.A.
Aspergillus nidulans A28	N.A.
Aspergillus fumigatus YJ-407	N.A.
Aspergillus flavus	N.A.
Aspergillus niger	N.A.
Beauveria bassiana 252	N.A.
Metarhizium robatsii 2575	N.A.
Pichia pastoris X33	N.A.

Note: Lethal concentration (C_L) was determined by the inhibition-zone assay. "N.A." means "no activity" at 1.0 nmol peptide each well.
